# Race, *APOE* genotypes, and cognitive decline among middle-aged urban adults

**DOI:** 10.1186/s13195-021-00855-y

**Published:** 2021-06-30

**Authors:** May A. Beydoun, Jordan Weiss, Hind A. Beydoun, Sharmin Hossain, Ana I. Maldonado, Botong Shen, Michele K. Evans, Alan B. Zonderman

**Affiliations:** 1 Laboratory of Epidemiology and Population Sciences, NIH Biomedical Research Center, National Institute on Aging, IRP, 251 Bayview Blvd., Suite 100, Room #: 04B118, Baltimore, MD 21224 USA; 2 Department of Demography, University of California, Berkeley, Berkeley, CA USA; 3 Department of Research Programs, Fort Belvoir Community Hospital, Fort Belvoir, VA USA; 4 Department of Psychology, University of Maryland Baltimore County, Catonsville, MD USA

**Keywords:** Apolipoprotein E, Cognitive aging, Racial disparities

## Abstract

**Background:**

Associations of *Apolipoprotein* (*APOE*) *ε*2 or *ε*4 (*APOE*2 or *APOE*4) dosages with cognitive change may differ across racial groups.

**Methods:**

Longitudinal data on 1770 middle-aged White and African American adults was compiled from the Healthy Aging in Neighborhoods of Diversity across the Life Span (HANDLS 2004-2013) study. *APOE*2 and *APOE*4 dosages were the two main exposures, while v_1_ and annual rate of change in cognitive performance (between v_1_ and v_2_) on 11 test scores were the main outcomes of interest (v1: 2004–2009 and v2: 2009–2013). Mixed-effects linear regression models were conducted adjusting for socio-demographic, lifestyle, and health-related potential confounders. Race (African American vs. White) and sex within racial groups were main effect modifiers.

**Results:**

Upon adjustment for multiple testing and potential confounders, *APOE*4 allelic dosage was associated with faster decline on a test of verbal memory among Whites only (CVLT-List A: γ_12_ = − 0.363 ± 0.137, *p* = 0.008), but not among African Americans. In contrast, among African American women, APOE4 dosage was linked to slower decline on a test of attention (BTA: γ_12_ = + 0.106 ± 0.035, *p* = 0.002), while no association was detected among African American men. *APOE*2 and *APOE*4 dosages showed inconsistent results in other domains of cognition overall and across racial groups that did not survive correction for multiple testing.

**Conclusions:**

In conclusion, *APOE*4 dosage was associated with faster decline on a test of verbal memory among Whites only, while exhibiting a potential protective effect among African American women in the domain of attention. Further longitudinal studies are needed to replicate our race and sex-specific findings.

**Supplementary Information:**

The online version contains supplementary material available at 10.1186/s13195-021-00855-y.

## Introduction

Evidence supporting a direct association between the Apolipoprotein E ε4 allele (*APOE*4) and the risk for age-related cognitive decline is growing; *APOE*4 status (i.e., having 1 or 2 ε4 alleles vs. none) is among the most well-established genetic risk factors for late onset Alzheimer’s disease (AD) and for age-related cognitive decline [1–22]. Although this association is generally consistent, some studies indicated that *APOE*4 may in fact reduce the risk for adverse cognitive outcomes, others failed to detect an association, and yet others found this relationship only among dementia patients [7–10]. Furthermore, whether the association of *APOE*4 with cognitive decline is specific to certain domains of cognition, such as verbal memory, is still unclear as is whether socio-demographic factors such as race act as important effect modifiers in that relationship.

Previous studies have detected direct associations between *APOE*4 and impairment or decline on domains of episodic memory [5, 11], verbal fluency [12], executive functioning [13, 14], perceptual/psychomotor speed, and visuo-spatial skill [11, 16, 17], as well as global mental status [9, 20]. Effect modification by sex in the relationship between *APOE* and cognitive outcomes has been investigated in several cross-sectional and longitudinal studie s[5, 6, 12, 23–28]. Earlier meta-analysis suggested sex differences in the *APOE* genotype-Alzheimer’s disease (AD) association [29], but several experimental and neurobiological studies indicated that the impact of *APOE*4 on neurodegeneration was more tangible among women compared to men [27, 30]. The studies that have examined associations between *APOE* and cognitive change thus far have been conducted using samples comprised largely of participants of European ancestry. Thus, race may also play an important role in this association, given the distributional differences in the *APOE* genotype by race, particularly between individuals of European and African ancestries. Only few studies have examined these associations in diverse population to understand how they may vary across racial groups. Most studies focused on the outcomes of incident AD or single domain (or global) cognitive decline (e.g., [3, 15]). It is worth noting that the association between race and cognitive decline may be more reflective of race as a social construct as opposed to ancestry. This is not the case for *APOE* genotype, which is largely determined by race as an ancestry construct. Importantly, there is a gap in the literature as to which domains of cognition are most affected by *APOE* genotypes differentially by race, as well as by sex within each racial group. The association of *APOE ε*2 (*APOE*2) allelic dosage with cognition, generally found protective against cognitive decline, also remains under-studied [31–34] especially in terms of race- and sex effect modifications.

The objectives of the present study were to (i) evaluate the associations of *APOE*2 and *APOE*4 allelic dosages with cognitive performance and change over time and (ii) explore racial differentials in those associations. As a secondary objective, (iii) we examined sex differences in those associations, overall, and within each racial group. We used data from the Healthy Aging in Neighborhoods of Diversity across the Life Span (HANDLS) study which consisted of African American and White men and women with baseline ages 30–64 years [35]. The HANDLS study was uniquely designed to study health disparities and contains an extant battery of cognitive measures as well as *APOE* genotype information, rendering it useful for addressing our research questions.

## Methods

### Study design

Our sample was drawn from the HANDLS study, an ongoing prospective cohort study of socioeconomically diverse African American and White men and women in Baltimore, MD [35]. Baseline data collection took place from 2004 to 2009 and was conducted in two phases. Phase I consisted of information collected from screening, recruitment, and a household interview which included a 24-h dietary recall [35]. Phase II involved an in-person physical health assessment which included, for example, a complete physical examination, an electrocardiogram, and a detailed cognitive battery [35]. Participants were invited to participate in a follow-up in-person assessment between 2009 and 2013. In addition to physical health measures, the HANDLS investigators also collected clinical and molecular biomarkers that span multiple physiological systems [35]. Written informed consent was obtained for all participants. The HANDLS study was approved by the Institutional Review Board of the National Institutes of Health, National Institute of Environmental Health Sciences [35].

Vital status in HANDLS was ascertained through linkage to the National Death Index (NDI), National Center for Health Statistics. The HANDLS-linked records include underlying cause of death in addition to other conditions or causes of death listed on the death certificate classified using the International Statistical Classification of Diseases, Version 10 (ICD-10) codes. Vital status information for all participants is available from enrollment (2004–2009) through December 31, 2018.

**Fig. 1:**
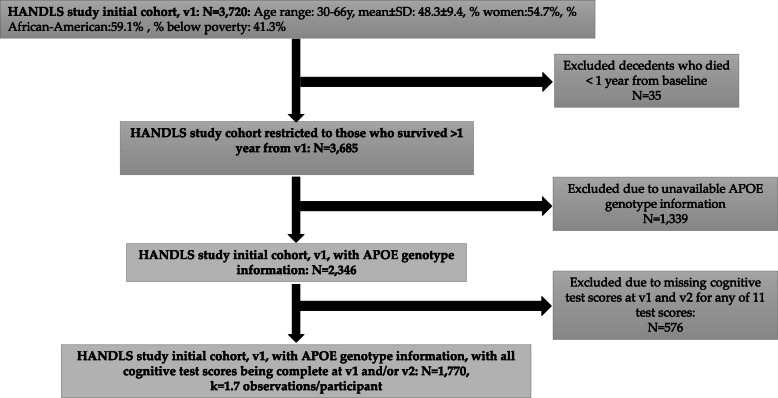
Participant flowchart. *Abbreviations*: APOE = Apolipoprotein E; HANDLS = Healthy Aging in Neighborhoods of Diversity across the Life Span

In this study, we utilized up to two repeats on cognitive test scores from v_1_ and/or v_2_ along with exposure data on *APOE* genotypes available for a sub-sample of Whites and African Americans participating in HANDLS, while excluding individuals who did not survive within a year of follow-up. Specifically, among 3720 initially recruited participants, we excluded those who died within 1 year of their baseline interview (*n* = 35) in the main analysis to ensure at least 12 months of follow-up; and then further excluded 1339 individuals for whom *APOE* genotype information was unavailable resulting in an analytic sample of 2346 individuals. Finally, we excluded 576 participants with missing or non-credible cognitive test information at both visits for all tests, which yielded an analytic sample of 1770 individuals, with an average number of observations/participant (k = 1.7), indicating 15% missingness on cognitive test performance outcomes (Fig. 1).

The inclusion criteria resulted in significant differences in characteristics of the samples relative to the complete baseline sample (*n* = 3720). In fact, individuals selected into the sample with complete cognitive test scores, *APOE* genotype exposures, and other non-covariate exclusions (*n* = 1770) were slightly older (mean [standard error]: 48.5 [0.22] vs 47.9 [0.22], *p = 0.0308*), more likely to be female (57.2% vs 52.6%, *p = 0.006*), less likely to be below poverty (38.9% vs 43.3%, *p* = 0.006), and more likely to be White (44.9% vs 37.4%, *p < 0.001*) than those excluded.

### Outcomes: cognitive measures

HANDLS researchers assessed cognitive function using a battery of tests which included the following: the Mini-Mental State Examination (MMSE), California Verbal Learning Test–List A (CVLT-List-A); California Verbal Learning Test–Free Recall Long Delay (CVLT-DFR), Benton Visual Retention Test (BVRT), Brief Test of Attention (BTA), Animal Fluency Test (AF), Digits Span Forward Test (DS-F), Digits Span Backward Test (DS-B), Clock Draw Test (CDT), Trailmaking Test A (Trails A), and Trailmaking Test B (Trails B). MMSE total score was considered as an outcome both in its initial scale and as normalized by using previously described methods [36]. Detailed description of each cognitive test score and the related domains are available as supplemental information (Method S1).

### Exposures: *APOE* allelic dosages

The *APOE* gene is coded by three common alleles (ε2, ε3, ε4) which form six genotypes (ε2/ε2, ε2/ε3, ε2/ε4, ε3/ε3, ε3/ ε4, ε4/ε4). In the current study, *APOE* genotype was determined on the basis of two variants (rs429358 [APOE-C112R], rs7412) [APOE-R158C]). Genotyping of these variants was achieved with the Taqman Assays (Applied Biosystems Assay-On-Demand part numbers C__3084793_20 and C__904973_10) on a 7900HT Sequence Detection System (Applied Biosystems). Detailed information on assay procedures are available elsewhere [37]. We classified *APOE* allelic dosage separately for ε2 and ε4 on the basis of having zero, one, or two of the aforementioned alleles. In both cases, the ε3/ε3 genotype acquired a value of zero. For *APOE*2 dosage, ε3/ε4 and ε4/ε4, also acquired a value of zero. For *APOE*4 dosage, ε2/ε2 and ε2/ε3 were also given a value of zero. *APOE*2 dosage had a value of 2 for ε2/ε2, while *APOE*4 dosage had a value of 2 for ε4/ε4. Thus, a value of 1 was ascribed to *APOE*2 dosage for the ε2/ε3 or ε2/ε4 genotypes, while a value of 1 was ascribed to *APOE*4 dosage for ε2/ε4 or ε3/ε4 genotypes.

### Covariates

This study considered among potential confounders several covariates for their documented association with cognitive performance or decline, which may also be associated with *APOE*2 or APOE4 dosage exposures. These included age at v1 (continuous, years), sex (male, female), race (White, African American), poverty status (below vs. above 125% the federal poverty line), educational attainment (less than high school, high school, more than high school), and literacy (Wide Range Achievement Test, third edition [WRAT-3]). Age at v_2_ was also used to compute time between visits 1 and 2. Poverty status was categorized by using the US Census Bureau poverty thresholds for 2004 relying on self-reported household income and total family size including children under age 18 years, with < 125% of the 2004 Health and Human Services poverty guidelines labelled as “below poverty” and ≥ 125% as “above poverty” [38]. Additionally, lifestyle and health-related factors were considered among potentially confounding covariates. Those measures included illicit drug use (0 = No vs. 1 = Yes, using any of marijuana, opiates, and cocaine), current smoking status (0 = No vs. 1 = Yes), body mass index (BMI, weight/height^2^ , kg m^−2^, continuous), self-rated health status coded 0 = poor/average (referent), 1 = good and 2 = very good/excellent, the Healthy Eating Index 2010 (HEI-2010), a measure of food and macronutrient-based overall diet quality, total energy intake (kcal/day), and the total score on the 20-item Center for Epidemiological Studies-Depression scale (CES-D), reflecting depressive symptoms. Furthermore, we accounted for an unweighted co-morbidity index composed of hypertension (0 = no, 1 = yes), diabetes (0 = diabetic, 1 = pre-diabetic, 2 = diabetic) and dyslipidemia (or statin use) (0 = no, 1 = yes), and self-reported history of any of several cardiovascular disease conditions (0 = no, 1 = yes). The latter component accounted for the occurrence of any of atrial fibrillation, angina, coronary artery disease, congestive heart failure, and myocardial infarction. Thus, the index’s potential range was between 0 and 5. Many of these measures, including poverty status and HEI-2010 are detailed elsewhere [39].

### Statistical analyses

We used Stata release 16 [40] to conduct all analyses. We described the selected sample at baseline, by utilizing means and proportions, and using *t*-tests to examine racial differences in those characteristics. To examine racial differences in continuous, binary, and categorical multi-level covariates, we used linear, logistic, multinomial logit, and by conducting linear mixed-effect models with *TIME* on study to examine the relationship between race and rate of change in cognitive performance. We ran both unadjusted models, and models adjusted for age, sex, and poverty status (Method S2). In terms of the main hypotheses, we tested cross-sectional and longitudinal associations between *APOE* allelic dosages and cognitive performance over time (i.e., baseline performance and annual rates of change), by conducting multiple mixed-effects linear regression models (Method S2), with *TIME* on study being considered the underlying time scale. Random effects were added to the intercept and the *TIME* variable, which was interacted with *APOE* allelic dosages (ε2 or ε4), as well as with all potentially confounding covariates, to test the covariate-adjusted associations of *APOE* allelic dosages with annual rates of change in cognitive performance. The main effects of *APOE*2 or *APOE*4 dosage exposures were included in the model—as were the main effects of other covariates—to examine the covariate-adjusted association of *APOE*2 or *APOE*4 dosage exposures with baseline cognitive performance. Modeling consisted of first fitting a minimally adjusted model (Model 1), with covariates included being baseline age, sex, race, and poverty status. Model 2 adjusted for all other socio-demographic, lifestyle, and health-related factors listed in the “Covariates” section, some of which could be considered as potential mediators. Nevertheless, we chose covariates related with cognitive performance trajectories in prior research. Due to missing data on many of these covariates (< 5% missing, on average) and to preserve sample size between reduced and full models, multiple imputations were carried out (5 imputations, 10 iterations) using the chained equations method. All covariates were utilized simultaneously in this estimation process, as was done in previous studies and continuous covariates were centered to their means [39]. Thus, Models 1 and 2 were applied to 2 exposures (*APOE*2 and *APOE*4 allelic dosages), 11 cognitive test scores (v_1_ cognitive performance and cognitive performance change over time), one main stratifying variable (race), and a secondary stratifying variable (sex within each racial group). Racial differences in the association between *APOE* allelic dosages and cognitive performance at v_1_ was tested using *APOE*2 or *APOE*4 × Race interaction terms in separate models, while that of the association between *APOE* allelic dosages and cognitive change was carried out by testing the *APOE*2 or *APOE*4 × TIME × Race term in the same model. In order to assess sex differences within each racial group, other models were conducted whereby sex was interacted with *APOE*2 or *APOE*4 and *APOE*2 or *APOE*4 × TIME among Whites and African Americans, separately. Sex differences overall were also assessed using a similar approach with 2-way and 3-way interaction terms (with *APOE* dosage exposures and *TIME*) and with sex instead of race.

We used a two-stage Heckman selection strategy for the mixed-effects linear regression models, thus partially accounting for sample selectivity. To this end, we first regressed an indicator of selection on age at baseline, sex, race, and poverty status using a probit model; this yielded an inverse mills ratio (IMR). At a second stage, we estimated our mixed-effects regression models adjusted for the IMR in addition to aforementioned covariates [39].

This study set the type I error rate a priori for main and interactive effects before correction for multiple testing to 0.05 and 0.10, respectively [41]. We accounted for outcome multiplicity (i.e., 11 cognitive test scores) using the approach of familywise Bonferroni correction [42], specifically for Model 1. As such, Model 2 was considered a sensitivity model in which potentially confounding and/or mediating factors were added. In this context, we adjusted significance levels for main effects to *p* < 0.0045 (0.05/11). Significance levels for the two-way interaction terms were adjusted to 0.10/11 = 0.009, as was done in previously published work [39], given the reduced statistical power of 2-way interactions [41]. The main findings were also presented as predictive margins of cognitive performance outcomes across follow-up time, within each APOE2 or APOE4 stratum and by race or race by sex. Standardized regression coefficients (“b”) for the APOE by TIME parameter was also presented and interpreted as the number of SD of cognitive performance change (for each group, i.e., race, sex, or sex by race), per 1 year change in follow-up time and 1 dosage increase in the APOE exposure. This measure can be extrapolated to a 10-year follow-up period for ease of interpretation. Given that the outcome is standardized, this can be used as a measure of effect size over a 10-year follow-up period. A sensitivity analysis was also conducted for all eligible participants, including those who died within 12 months of follow-up.

## Results

Table 1 displays study sample characteristics, overall, and by race in the final analytic sample. Overall, the vast majority of participants were non-ε2 (80.5%) and/or non-ε4 (66.7%) allele carriers. African Americans were more likely than Whites to be *APOE*4 carriers, including the ε4/ε4 genotype, a pattern also observed for *APOE*2 carrier status, specifically for the *APOE*2 dosage of 1. In fact, the ε3/ε3 genotype was significantly more common in Whites vs. African Americans (61.1% vs. 43.0%, *p* < 0.05). African Americans had a higher likelihood than Whites to be living below poverty (45% vs. 31%), coupled with a lower percentage above HS level of education (34% vs. 36%). They also had a lower mean for WRAT-3 literacy score (40.8 vs. 44.8 among Whites, *p* < 0.05). Racial differences, though inconsistent in terms of directionality, were also detected with respect to current drug use, self-rated health, depressive symptoms, hypertension, dyslipidemia, and cardiovascular disease. Cognitive test scores were generally suggestive of poorer performance among African Americans as opposed to Whites. Nevertheless, the pace of decline among African Americans was faster than among Whites only in the case of BVRT, and independently of age, sex, and poverty status. Furthermore, time on study among those with complete visit 2, differed significantly by race, with African Americans having a longer mean follow-up time compared to Whites (Mean ± SE, years: 4.78 ± 0.04 vs. 4.29 ± 0.03, *p* < 0.001).

**Table 1:** Study sample characteristics, overall, and by race in final analytic sample with imputed covariates (*N* = 1770), HANDLS 2004–2013

	Overall	Whites	African American
	(X ± SE), %	(X ± SE), %	(X ± SE), %
	(***N*** = 1770)	(***N*** = 794)	(***N*** = 976)
*APOE* genotype, %			
ε3/ε3	51.1 ± 1.2	61.1 ± 1.7	43.0 ± 1.6
ε2/ε2	0.7 ± 0.2	0.5 ± 0.3	0.9 ± 0.3
ε2/ε3	14.9 ± 0.8	12.6 ± 1.2^***d^	16.7 ± 1.2
ε2/ε4	4.0 ± 0.5	2.4 ± 0.5^***d^	5.2 ± 0.7
ε3/ε4	25.4 ± 1.0	21.2 ± 1.4^***d^	28.9 ± 1.5
ε4/ε4	3.9 ± 0.5	2.3 ± 0.5^***d^	5.2 ± 0.7
*APOE2* allelic dosage			
0	80.5 ± 0.9	84.5 ± 1.3	77.2 ± 1.3
1	18.8 ± 0.9	15.0 ± 1.3^***d^	21.9 ± 1.3
2	0.7 ± 0.2	0.5 ± 0.3	0.9 ± 0.3
X ± SE			
*APOE4* allelic dosage			
0	66.7 ± 1.1	74.2 ± 1.6	60.7 ± 1.6
1	29.4 ± 1.1	23.6 ± 1.5^***d^	34.1 ± 1.5
2	3.9 ± 0.5	2.3 ± 0.5^***d^	5.2 ± 0.7
X ± SE			
Baseline socio-demographic, SES, and health-related variables			
Sex, % male	42.8 ± 1.2	43.7 ± 1.8	42.1 ± 1.6
Age at v_1_, years	48.496 ± 0.218	48.606 ± 0.325	48.406 ± 0.294
African American, %	55.1 ± 1.2	0.000	100.0
Poverty status, % < 125% of the 2004 federal poverty guidelines	38.9 ± 1.2	31.0 ± 1.6^***^	45.3 ± 1.6
Education, completed, %			
< HS	5.9 ± 0.6	8.5 ± 1.0^***d^	3.9 ± 0.6
HS	59.1 ± 1.2	55.9 ± 1.8	61.9 ± 1.6
> HS	34.8 ± 1.1	35.7 ± 1.7	34.2 ± 1.5
Literacy, WRAT-3 score	42.6 ± 0.2	44.8 ± 0.3^***d^	40.8 ± 0.2
Baseline drug and tobacco use			
Any drug, current user, %	17.5 ± 0.9	13.1 ± 1.2^***d^	21.0 ± 1.4
Tobacco, current user, %	44.8 ± 1.2	43.1 ± 1.8	46.2 ± 1.6
Body mass index, kg/m^2^	30.1 ± 0.2	30.3 ± 0.3	30.0 ± 0.3
Self-rated health, %			
Poor/average	24.1 ± 1.0	26.7 ± 1.6^**d^	21.9 ± 1.3
Good	41.7 ± 1.2	37.5 ± 1.7	45.2 ± 1.6
Very good/excellent	34.2 ± 1.1	35.8 ± 1.7^*d^	32.9 ± 1.5
HEI-2010 total score at v_1_	42.5 ± 0.3	42.2 ± 0.5	42.8 ± 0.4
Total energy intake, kcal/day	2018 ± 24	2035 ± 38	2003 ± 35
CES-D total score	14.9 ± 0.3	15.5 ± 0.4^*d^	14.4 ± 0.4
Hypertension^a^, %	46.1 ± 1.2	40.3 ± 1.8^***d^	50.8 ± 1.6
Diabetes^a^, %			
No	64.3 ± 1.1	63.3 ± 1.7	65.1 ± 1.5
Pre-diabetic	18.6 ± 0.9	20.8 ± 1.5	16.9 ± 1.2
Diabetic	17.1 ± 0.9	16.0 ± 1.3	18.1 ± 1.2
Dyslipidemia^a^, %	28.8 ± 1.1	33.6 ± 1.8^***d^	24.9 ± 1.5
Cardiovascular disease^a^, %	17.6 ± 0.9	15.2 ± 1.3*	19.7 ± 1.3
Co-morbidity index^a^	3.46 ± 0.034	3.43 ± 0.05	3.48 ± 0.05
Cognitive performance at v_1_, unadjusted^b^			
MMSE, non-normalized	27.772 ± 0.051	28.163 ± 0.075^***d^	27.454 ± 0.069
MMSE, normalized	76.7 ± 0.37	80.3 ± 0.56^***d^	73.8 ± 0.48
CVLT-List A	24.635 ± 0.167	26.044 ± 0.263^***d^	23.541 ± 0.207
CVLT-DFR	7.321 ± 0.079	8.131 ± 0.121^***d^	6.690 ± 0.099
BVRT	6.339 ± 0.118	6.062 ± 0.167^*^	6.566 ± 0.166
BTA	6.692 ± 0.055	7.199 ± 0.079^***d^	6.287 ± 0.072
AF	18.866 ± 0.127	19.776 ± 0.198^***d^	18.125 ± 0.160
DS-F	7.317 ± 0.052	7.633 ± 0.081^***d^	7.058 ± 0.067
DS-B	5.692 ± 0.052	6.234 ± 0.083^***d^	5.246 ± 0.063
CDT	8.799 ± 0.029	8.972 ± 0.041^***d^	8.656 ± 0.039
TRAILS A	36.392 ± 0.915	31.690 ± 0.831^***d^	40.246 ± 1.508
TRAILS B	143.723 ± 3.702	111.639 ± 4.573^***d^	170.019 ± 5.455
Annualized rate of cognitive change, unadjusted^c^			
MMSE, non-normalized	− 0.0116 ± 0.0103	− 0.0055 ± 0.0149	− 0.0101 ± 0.0141
MMSE, normalized	− 0.1941 ± 0.0847^┼^	− 0.1596 ± 0.1315	− 0.1570 ± 0.1111
CVLT-List A	− 1.353 ± 0.0430^┼^	− 1.4380 ± 0.0719^┼^	− 1.2940 ± 0.05259^┼^
CVLT-DFR	− 0.4505 ± 0.0179^┼^	− 0.4804 ± 0.0296^┼^	− 0.4258 ± 0.02214^┼^
BVRT	+ 0.4659 ± 0.0266^┼^	+ 0.3166 ± 0.0358^┼,***,d^	+ 0.5669 ± 0.0375^┼^
BTA	− 0.0618 ± 0.01243^┼^	− 0.0803 ± 0.0192^┼^	− 0.0447 ± 0.0163^┼^
AF	+ 0.0132 ± 0.0242	+ 0.0400 ± 0.0402	+ 0.0025 ± 0.0298
DS-F	− 0.0139 ± 0.0102	− 0.0008 ± 0.0169	− 0.0196 ± 0.0128
DS-B	− 0.0067 ± 0.0103	+ 0.0071 ± 0.0164	− 0.0113 ± 0.0132
CDT	− 0.0155 ± 0.0075^┼^	− 0.0200 ± 0.0115	− 0.00958 ± 0.00990
TRAILS A	+ 0.4719 ± 0.2897	+ 0.3703 ± 0.2800	+ 0.3995 ± 0.4610
TRAILS B	+ 4.0767 ± 0.8128^┼^	+ 2.298 ± 1.015^┼^	+ 5.0957 ± 1.1905^┼^

Abbreviations*: AF* animal fluency, *APOE* Apolipoprotein E genotype, *BMI* body mass index, *BTA* Brief Test of Attention, *BVRT* Benton Visual Retention Test, *CDT* Clock Drawing Test, *CES-D* Center for Epidemiologic Studies-Depression, *CVLT-DFR* California Verbal Learning Test-Delayed Free Recall, *CVLT-List A* California Verbal Learning Test-List A, *DS-B* Digits Span-Backward, *DS-F* Digits Span-Forward, *HANDLS* Healthy Aging in Neighborhood of Diversity across the Lifespan, *HEI-2010* Healthy Eating Index, 2010 version, *HS* High school, *MMSE* Mini-Mental State Examination, *SD* standard deviation, *TRAILS A* Trailmaking Test, Part A, *TRAILS B* Trailmaking Test, part B, *WRAT-3* Wide Range Achievement Test, 3rd revision, *X* mean

Values are means (X) ± SE for continuous variables and % for categorical variables. The sample selected has complete data on MMSE and 10 other cognitive test scores at visits 1 and/or 2 and complete data on *APOE* genotypes. Other covariates were multiple imputed (5 imputations with 10 iterations), using chained equations. All cognitive test scores are in the direction of higher score ➔ better performance with the exception of BVRT (# of errors) and TRAILS A and B (# of sec. to complete)

^a^The co-morbidity index was calculated as the sum of hypertension, diabetes, and dyslipidemia (or statin use), and self-reported history of cardiovascular disease included atrial fibrillation, angina, coronary artery disease, congestive heart failure, or myocardial infarction, ranging from 0 to 5

^b^Crude baseline cognitive test score

^c^Crude estimated annual rate of change in cognitive performance based on mixed-effects linear regression model with TIME as the only covariate. Difference by race was determined by interacting *TIME* with race

^d^*p* < 0.05 upon further adjustment for age, sex, and poverty status in multiple linear, logistic, multinomial logit, or mixed-effects linear regression models with race entered as the main predictor

*p < 0.05; ** *p* < 0.01; *** *p* < 0.001, *t*-test for null hypothesis of no between-race differences

^┼^*p* < 0.05, *t*-test for null hypothesis of γ_1_ = 0 (fixed effects coefficient for *TIME*) in mixed-effects linear regression models with *TIME* as the only variable

**Fig. 2:**
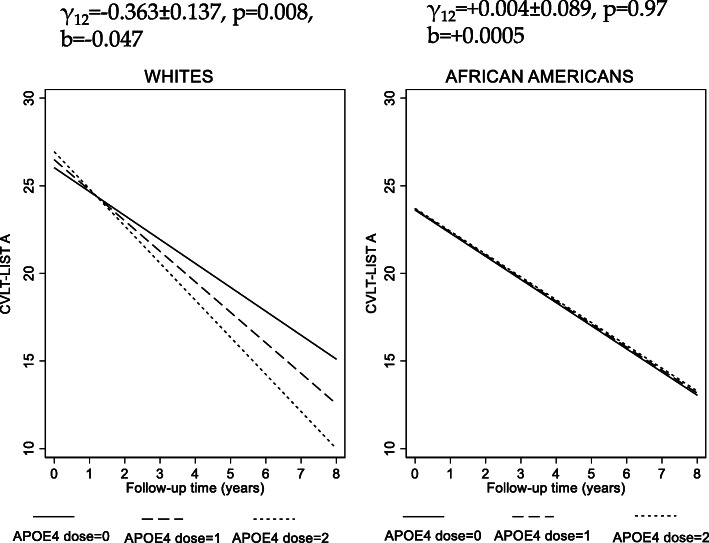
APOE4 allelic dosage vs. CVLT-List A trajectories among Whites and African Americans: predictive margins from mixed-effects linear regression model^a^. *Abbreviations*: APOE = Apolipoprotein E; CVLT-List A = California Verbal Learning Test, List A; HANDLS = Healthy Aging in Neighborhoods of Diversity across the Life Span. ^a^ Model 2B, Table 2, fully adjusted model, stratified by race. Figure shows predictive margins of CVLT-List A scores across time, highlighting the significant difference in slope across APOE4 dosage among Whites only. γ_12_ refers to the fixed effect of APOE4 dosage on the rate of change in CVLT-List A total score for each racial group. “b” is a standardized measure for the regression coefficient γ_12_ in model where CVLT-LIST A is entered as standardized z-scores within each racial group. It is interpreted as the SD of outcome change per year increase in follow-up time for each APOE4 dosage change. 1 SD of CVLT-LIST A corresponds to 7.71 change in score, overall

Table 2 presents the findings from a series of linear mixed-effects regression models examining the associations of *APOE*2 or *APOE*4 allelic dosages with cognitive performance over time. These associations were tested overall and by race, and heterogeneity by sex was also tested within each racial category. After correction for multiple testing, most of the associations were deemed nonsignificant. Only one association passed the familywise Bonferroni correction criterion. The latter indicated that *APOE*4 dosage was associated with faster decline on a test of verbal memory, immediate recall among Whites only (CVLT-List A, *TIME* × *APOE*4, among Whites, γ_12_ = − 0.370 ± 0.139, p = 0.008), in the minimally adjusted model (i.e., Model 1). This association remained virtually unaltered after further adjustment for additional socio-demographic, lifestyle, and health-related factors (γ_12_ = − 0.364 ± 0.137, p = 0.008) and was significantly different between Whites and African Americans (CVLT-List A, *TIME*×*APOE*4 × Race, overall sample, *p* < 0.05). A marginal association (*P* < 0.05) in the same direction that did not pass correction for multiple testing was also found between *APOE*4 and CVLT-DFR annual rate of change, also among Whites only with a significant difference by race, indicating an adverse effect of *APOE*4 dosage on memory decline within that racial group in particular. This association was also unaltered by additional adjustment for important potential confounders. The difference in trajectories of CVLT-List A across *APOE*4 dosages among Whites and African Americans are shown in Fig. 2. These figures include a measure of standardized regression coefficient for the annualized rate of change in CVLT-List A, whereby the cognitive outcome was entered as a standardized z-score. This effect can be interpreted as a − 0.50 SD difference in CVLT-List A score after change a 10-year follow-up period (b = 0.050 for a 1-year follow-up), contrasting APOE4 dosages of “1” vs. “0” or “2” vs. “1,” among Whites. This effect size is reduced to + 0.005 SD among African Americans after 10 years of follow-up. All SDs for 11 cognitive performance tests over the period of follow-up (v1 through v2), and in the overall eligible sample (*N* = 1770), are presented in Figure S1 to contextualize the effects observed of APOE exposures on cognitive performance and change in the mixed-effects linear regression models.

**Table 2:** *APOE*2 and *APOE*4 allelic dosages and their association with cognitive performance at v1 and change over time: overall and race-specific mixed-effects linear regression models: HANDLS 2004–2013

	*APOE2* allelic dosage		*APOE4* allelic dosage	
	Model 1	Model 2	Model 1	Model 2
	*γ* ± SE	*γ* ± SE	*γ* ± SE	*γ* ± SE
**Overall**	**(*N*** **= 1770, k = 1.7)**	**(*N*** **= 1770, k = 1.7)**	**(*N*** **= 1770, k = 1.7)**	**(*N*** **= 1770, k = 1.7)**
*Outcome = Cognitive performance test score*				
Normalized MMSE				
Exposure, *γ*_*0a*_	− 0.652 ± 0.813	− 0.129 ± 0.696	+ 0.041 ± 0.616	− 0.082 ± 0.528
Exposure × TIME, *γ*_*1a*_	− 0.394 ± 0.202*	− 0.429 ± 0.196*	+ 0.190 ± 0.153	+ 0.175 ± 0.149
CVLT-List A				
Exposure, *γ*_*0a*_	− 0.290 ± 0.369	− 0.150 ± 0.343	+ 0.151 ± 0.282	+ 0.121 ± 0.263
Exposure × TIME, *γ*_*1a*_	+ 0.081 ± 0.100	+ 0.054 ± 0.099	− 0.111 ± 0.076 ^b^	− 0.121 ± 0.076 ^b^
CVLT-DFR				
Exposure, *γ*_*0a*_	− 0.211 ± 0.173	− 0.164 ± 0.163	− 0.001 ± 0.132	− 0.037 ± 0.125
Exposure × TIME, *γ*_*1a*_	+ 0.081 ± 0.041	+ 0.073 ± 0.041	− 0.025 ± 0.032 ^b^	− 0.024 ± 0.032 ^b^
BVRT				
Exposure, *γ*_*0a*_	− 0.167 ± 0.269	− 0.203 ± 0.250	+ 0.359 ± 0.203	+ 0.376 ± 0.190*
Exposure × TIME, *γ*_*1a*_	+ 0.066 ± 0.062	+ 0.058 ± 0.062	− 0.020 ± 0.047	− 0.018 ± 0.047
BTA				
Exposure, *γ*_*0a*_	+ 0.011 ± 0.122	+ 0.039 ± 0.116	− 0.094 ± 0.092	− 0.079 ± 0.087
Exposure × TIME, *γ*_*1a*_	+ 0.007 ± 0.029	+ 0.007 ± 0.029	+ 0.031 ± 0.022 ^b^	+ 0.029 ± 0.022 ^b^
AF				
Exposure, *γ*_*0a*_	− 0.053 ± 0.292	+ 0.041 ± 0.276	+ 0.094 ± 0.221	+ 0.044 ± 0.209
Exposure × TIME, *γ*_*1a*_	− 0.063 ± 0.057	− 0.064 ± 0.057	− 0.001 ± 0.043	− 0.001 ± 0.044
DS-F				
Exposure, *γ*_*0a*_	− 0.037 ± 0.121	+ 0.001 ± 0.110	− 0.004 ± 0.092	− 0.024 ± 0.084
Exposure × TIME, *γ*_*1a*_	+ 0.005 ± 0.024	+ 0.007 ± 0.024	+ 0.039 ± 0.018*	+ 0.038 ± 0.018*
DS-B				
Exposure, *γ*_*0a*_	− 0.093 ± 0.119	− 0.051 ± 0.106	0.054 ± 0.090	+ 0.043 ± 0.080
Exposure × TIME, *γ*_*1a*_	− 0.002 ± 0.024	− 0.003 ± 0.024	0.005 ± 0.019	+ 0.001 ± 0.018
CDT				
Exposure, *γ*_*0a*_	+ 0.011 ± 0.067	+ 0.017 ± 0.065	− 0.039 ± 0.051	− 0.042 ± 0.050
Exposure × TIME, *γ*_*1a*_	− 0.011 ± 0.018	− 0.011 ± 0.018	− 0.009 ± 0.014	− 0.010 ± 0.014
TRAILS A				
Exposure, *γ*_*0a*_	− 1.876 ± 2.248	− 1.626 ± 2.237	+ 0.699 ± 1.700	+ 0.702 ± 1.694
Exposure × TIME, *γ*_*1a*_	0.685 ± 0.688	+ 0.643 ± 0.689	− 0.191 ± 0.524	− 0.261 ± 0.525
TRAILS B				
Exposure, *γ*_*0a*_	+ 2.899 ± 8.257	+ 1.548 ± 7.698	+ 2.330 ± 6.243 ^a^	+ 2.434 ± 5.826^a^
Exposure × TIME, *γ*_*1a*_	− 1.362 ± 1.922	− 1.570 ± 1.925	0.110 ± 1.456 ^b^	+ 0.130 ± 1.461^b^
**Whites**	**(N = 794, k = 1.8)**	**(N = 794, k = 1.7)**	**(N = 794, k = 1.8)**	**(N = 794, k = 1.7)**
*Outcome = Cognitive performance test score*				
Normalized MMSE				
Exposure, *γ*_*0a*_	− 0.160 ± 1.368	− 0.390 ± 1.112	+ 0.371 ± 1.043	+ 0.334 ± 0.853
Exposure × TIME, *γ*_*1a*_	− 0.402 ± 0.356	− 0.366 ± 0.344	+ 0.291 ± 0.260^d^	+ 0.337 ± 0.254
CVLT-List A				
Exposure, *γ*_*0a*_	+ 0.528 ± 0.645	+ 0.442 ± 0.592	+ 0.414 ± 0.495	+ 0.446 ± 0.454
Exposure × TIME, *γ*_*1a*_	+ 0.062 ± 0.192	+ 0.047 ± 0.189	− 0.370 ± 0.139**	− 0.363 ± 0.137**
CVLT-DFR				
Exposure, *γ*_*0a*_	+ 0.115 ± 0.297	+ 0.074 ± 0.278	+ 0.070 ± 0.228	+ 0.080 ± 0.213
Exposure × TIME, *γ*_*1a*_	+ 0.103 ± 0.078	+ 0.105 ± 0.077	− 0.127 ± 0.057*	− 0.115 ± 0.056*
BVRT				
Exposure, *γ*_*0a*_	+ 0.121 ± 0.421	+ 0.218 ± 0.364	+ 0.346 ± 0.321	+ 0.291 ± 0.278
Exposure × TIME, *γ*_*1a*_	+ 0.090 ± 0.098	+ 0.106 ± 0.097	+ 0.053 ± 0.071	+ 0.044 ± 0.071
BTA				
Exposure, *γ*_*0a*_	− 0.041 ± 0.197	− 0.062 ± 0.186	− 0.086 ± 0.147	− 0.050 ± 0.139
Exposure × TIME, *γ*_*1a*_	0.040 ± 0.051^d^	+ 0.024 ± 0.050^d^	− 0.043 ± 0.038	− 0.043 ± 0.037
AF				
Exposure, *γ*_*0a*_	+ 0.562 ± 0.507	+ 0.460 ± 0.461	+ 0.212 ± 0.387	+ 0.263 ± 0.350
Exposure × TIME, *γ*_*1a*_	− 0.065 ± 0.108	− 0.083 ± 0.110	− 0.032 ± 0.079	− 0.026 ± 0.080
DS-F				
Exposure, *γ*_*0a*_	− 0.038 ± 0.207	− 0.099 ± 0.181	− 0.198 ± 0.158	− 0.215 ± 0.137
Exposure × TIME, *γ*_*1a*_	− 0.004 ± 0.045	− 0.010 ± 0.046	0.046 ± 0.033	+ 0.047 ± 0.034
DS-B				
Exposure, *γ*_*0a*_	− 0.100 ± 0.211	− 0.172 ± 0.181	+ 0.045 ± 0.162	+ 0.041 ± 0.139
Exposure × TIME, *γ*_*1a*_	+ 0.003 ± 0.044	+ 0.002 ± 0.043	+ 0.022 ± 0.033	+ 0.015 ± 0.032
CDT				
Exposure, *γ*_*0a*_	− 0.021 ± 0.105	− 0.050 ± 0.104	− 0.022 ± 0.083	− 0.011 ± 0.080
Exposure × TIME, *γ*_*1a*_	− 0.039 ± 0.031^d^	− 0.043 ± 0.031	− 0.038 ± 0.023	− 0.032 ± 0.023
TRAILS A				
Exposure, *γ*_*0a*_	− 1.800 ± 2.293	− 1.139 ± 2.250	2.521 ± 1.751	+ 2.428 ± 1.715
Exposure × TIME, *γ*_*1a*_	+ 0.096 ± 0.755	+ 0.095 ± 0.754	− 0.379 ± 0.556	− 0.491 ± 0.555
TRAILS B				
Exposure, *γ*_*0a*_	+ 3.770 ± 11.488	+ 7.185 ± 10.406	+ 19.112 ± 8.730*	+ 19.258 ± 7.937*
Exposure × TIME, *γ*_*1a*_	− 3.513 ± 2.701	− 3.187 ± 2.663	− 4.140 ± 2.000*	− 4.408 ± 1.981*
**African American**	**(*N*** **= 976, k = 1.8)**	**(*N*** **= 976, k = 1.8)**	**(*N*** **= 976, k = 1.8)**	**(*N*** **= 976, k = 1.8)**
*Outcome = Cognitive performance test score*				
MMSE				
Exposure, *γ*_*0a*_	− 1.094 ± 1.005	− 0.079 ± 0.894	− 0.09 ± 0.758	− 0.274 ± 0.675
Exposure × TIME, *γ*_*1a*_	− 0.360 ± 0.246	− 0.432 ± 0.242	+ 0.139 ± 0.190	+ 0.110 ± 0.186
CVLT-List A				
Exposure, *γ*_*0a*_	− 0.767 ± 0.437	− 0.472 ± 0.413	+ 0.077 ± 0.335	+ 0.044 ± 0.316
Exposure × TIME, *γ*_*1a*_	+ 0.104 ± 0.115	+ 0.073 ± 0.115	+ 0.016 ± 0.089	+ 0.004 ± 0.089
CVLT-DFR				
Exposure, *γ*_*0a*_	− 0.405 ± 0.209	− 0.314 ± 0.200	− 0.015 ± 0.160	− 0.022 ± 0.153
Exposure × TIME, *γ*_*1a*_	+ 0.078 ± 0.048	+ 0.072 ± 0.048	+ 0.027 ± 0.038	+ 0.020 ± 0.038
BVRT				
Exposure, *γ*_*0a*_	− 0.322 ± 0.348	− 0.423 ± 0.336	+ 0.293 ± 0.262	+ 0.342 ± 0.253
Exposure × TIME, *γ*_*1a*_	0.058 ± 0.083	+ 0.042 ± 0.083	− 0.053 ± 0.064	− 0.046 ± 0.064
BTA				
Exposure, *γ*_*0a*_	0.027 ± 0.156	+ 0.089 ± 0.150	− 0.088 ± 0.118	− 0.086 ± 0.114
Exposure × TIME, *γ*_*1a*_	− 0.005 ± 0.036	− 0.005 ± 0.036	+ 0.065 ± 0.028*,^d^	+ 0.064 ± 0.028*^,d^
AF				
Exposure, *γ*_*0a*_	− 0.383 ± 0.347	− 0.257 ± 0.338	+ 0.087 ± 0.262	+ 0.038 ± 0.255
Exposure × TIME, *γ*_*1a*_	− 0.061 ± 0.066	− 0.050 ± 0.066	+ 0.032 ± 0.054	+ 0.006 ± 0.051
DS-F				
Exposure, *γ*_*0a*_	− 0.044 ± 0.148	+ 0.021 ± 0.139	+ 0.117 ± 0.111	+ 0.095 ± 0.105
Exposure × TIME, *γ*_*1a*_	+ 0.004 ± 0.028	+ 0.007 ± 0.028	+ 0.033 ± 0.021	+ 0.032 ± 0.021
DS-B				
Exposure, *γ*_*0a*_	− 0.100 ± 0.139	− 0.014 ± 0.127	+ 0.060 ± 0.105	+ 0.045 ± 0.096
Exposure × TIME, *γ*_*1a*_	− 0.006 ± 0.029	− 0.009 ± 0.029	− 0.002 ± 0.022	− 0.006 ± 0.022
CDT				
Exposure, *γ*_*0a*_	+ 0.033 ± 0.086	+ 0.060 ± 0.084	− 0.041 ± 0.065	− 0.043 ± 0.064^c^
Exposure × TIME, *γ*_*1a*_	− 0.002 ± 0.022^d^	− 0.003 ± 0.022^d^	+ 0.005 ± 0.017^d^	+ 0.003 ± 0.017^d^
TRAILS A				
Exposure, *γ*_*0a*_	− 1.798 ± 3.475	− 1.611 ± 3.472	− 0.322 ± 2.610	− 0.167 ± 2.612
Exposure × TIME, *γ*_*1a*_	+ 1.004 ± 1.023	+ 0.985 ± 1.030	− 0.089 ± 0.790	− 0.100 ± 0.793
TRAILS B				
Exposure, *γ*_*0a*_	+ 3.643 ± 11.608	− 1.801 ± 10.944	− 7.672 ± 8.726	− 6.498 ± 8.229
Exposure × TIME, *γ*_*1a*_	− 0.761 ± 2.638	− 1.087 ± 2.651	+ 2.928 ± 2.162	+ 2.710 ± 2.021

Abbreviations*: AF* animal fluency, *APOE* Apolipoprotein E genotype, *BMI* body mass index, *BTA* Brief Test of Attention, *BVRT* Benton Visual Retention Test, *CDT* Clock Drawing Test, *CES-D* Center for Epidemiologic Studies-Depression, *CVLT-DFR* California Verbal Learning Test-Delayed Free Recall, *CVLT-List A* California Verbal Learning Test-List A, *DS-B* Digits Span-Backward, *DS-F* Digits Span-Forward, *HANDLS* Healthy Aging in Neighborhood of Diversity across the Lifespan, *HEI-2010* Healthy Eating Index, 2010 version, *HS* high school, *MMSE* Mini-Mental State Examination, *SD* Standard Deviation, *TRAILS A* Trailmaking Test, Part A, *TRAILS B* Trailmaking Test, part B, *WRAT-3* Wide Range Achievement Test, 3rd revision, *X* mean

Models 1A.1-1K included each of *APOE2* or *APOE4* allelic dosages, separately as the main predictor for v1 cognitive performance and cognitive change over time (11 test scores), using a series of mixed-effects linear regression models, carried out in the overall population and stratified by race. These models adjusted only for age, sex, race, poverty status, and the inverse mills ratio. Models 2A.1-2K followed a similar approach but adjusted further for selected socio-demographic, lifestyle, and health-related factors, namely educational attainment, the WRAT-3 score, current drug use, current tobacco use, body mass index, self-rated health, co-morbidity index, HEI-2010, total energy intake, and the CES-D total score

^a^ *p* < 0.05 for Race × (*APOE*2 or *APOE*4) in models that are unstratified by race to which this 2-way interaction was included

^b^ *p* < 0.05 for Race × (*APOE*2 or *APOE*4) × *TIME* in models that are unstratified by race to which this 3-way interaction was included

^c^ *p* < 0.05 for Sex × (*APOE*2 or *APOE*4) in models that are stratified by race to which this 2-way interaction was included

^d^ *p* < 0.05 for Sex × (*APOE*2 or *APOE*4) × *TIME* in models that are stratified by race to which this 3-way interaction was included

*p < 0.05; **p < 0.01, test for null hypothesis of γ = 0. Bolded values passed correction for multiple testing

Other important associations that were detected in race-stratified models (Table 2) prior to correction for multiple testing included an inverse association between *APOE*2 dosage and MMSE annual rate of change, overall, and in both models and a direct relationship between *APOE*4 dosage and BTA annual rate of change among African Americans (Models 1 and 2). Moreover, among Whites, TRAILS B performance at v_1_ was poorer with increased *APOE*4 dosage, while annual rate of change in TRAILS B with *APOE*4 dosage indicated an opposing trend (i.e., improvement over time). This was not the case among African Americans [TRAILS B (overall sample), *APOE*4 × Race, *APOE*4 × *TIME* × Race, *P* < 0.05].

Findings from the secondary analyses, stratified by sex, are shown in Table S1. The results indicate that there were no detected sex-specific associations between APOE2 or APOE4 dosages and cognitive performance at v_1_ or decline over time, after adjustment for multiple testing.

**Fig. 3:**
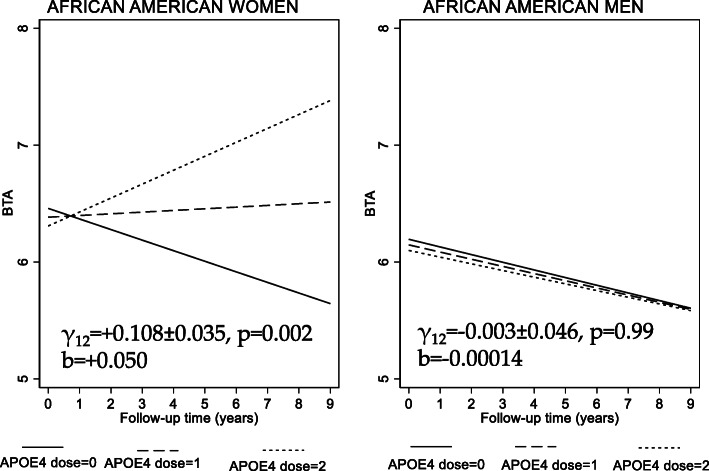
APOE4 allelic dosage vs. BTA trajectories among African American women and men: predictive margins from mixed-effects linear regression model^a^. *Abbreviations*: APOE = Apolipoprotein E; BTA = Brief Test of Attention; HANDLS = Healthy Aging in Neighborhoods of Diversity across the Life Span. ^a^ Model 2E, Table 2, fully adjusted model, stratified by race and sex. Figure shows predictive margins of BTA scores across time with their 95% CI, highlighting the significant difference in slope across APOE4 dosage among African American women only. γ_12_ refers to the fixed effect of APOE4 dosage on the rate of change in BTA total score for each sex group among African Americans. “b” is a standardized measure for the regression coefficient γ_12_ in model where BTA is entered as standardized z-scores within each racial/sex group. It is interpreted as the SD of outcome change per year increase in follow-up time for each APOE4 dosage change. 1 SD of BTA corresponds to 6.63 change in score, overall

Sex differences in associations of APOE2 or APOE4 allelic dosages with cognitive performance over time were noted within each racial group (Table 2 and S2). Among Whites, one association (*APOE*2 dosage vs. change in BTA over time) was more pronounced in women (*APOE*2 × TIME × Sex, *p* < 0.05). Specifically, among White women, *APOE*2 allelic dosage was directly associated with annual rate of change in BTA, reflecting a protective effect (γ_11_ = + 0.145 ± 0.070, p = 0.039, Model 1; γ_11_ = + 0.160 ± 0.069, *p* = 0.020, Model 2), an association not detected among White men. Among African Americans, several other associations between *APOE*2 or *APOE*4 dosages and cognitive performance over time differed between men and women. Most notably, *APOE*4 dosage was directly associated with annual rate of change in BTA, suggestive of improvement over time in the domain of attention, among African American women (γ_12_ = + 0.114 ± 0.035, p = 0.001, Model 1; γ_12_ = + 0.106 ± 0.035, *p* = 0.002, Model 2) and passing correction for multiple testing, although that relationship was not detected among men (*p* > 0.10). The contrast in BTA trajectories across *APOE*4 dosages based on Model 2 between African American women and men is displayed in Fig. 3. Similar to Figs. 2, the effect sizes are shown in Figs. 3, indicating that after a 10-year follow-up, a one dosage increase in APOE4 is associated with a + 0.50 SD (b = 0.050 for a 1 year follow-up) difference in change in BTA score among African American women, as opposed to a − 0.0014 SD difference in change in BTA among African American men after 10 years of follow-up (see Figure S1 for SDs of cognitive performance test scores).

Finally, among African American men (unlike among women), both *APOE*2 and *APOE*4 dosages were directly linked to annual rates of change in CDT, suggestive of improvement over time in the visuo-spatial abilities domain (*p* < 0.10 for γ_11_ and γ_12_ in Model 1), though these associations did not survive correction for multiple testing. They were also markedly attenuated in Model 2, mainly due to adjustment for current smoking and drug use status and HEI-2010 total score. Our key findings were not altered when a sensitivity analysis was conducted with all eligible participants, including those that died within 12 months of follow-up (*n* < 10 for each analysis).

## Discussion

### Summary of findings

Our present work is among few longitudinal studies to examine racial differences in the associations of *APOE*2 and *APOE*4 dosages with cognitive performance over time, particularly longitudinal change in test scores spanning key domains of cognition in sample of middle-aged urban adults. We observed several key findings. Upon correction for multiple testing, socio-demographic, lifestyle, and health-related potential confounders, *APOE*4 allelic dosage was associated with faster decline on a test of verbal memory among Whites only (CVLT-List A: γ_12_ = − 0.363 ± 0.137, p = 0.008), but not among African Americans. In contrast, among African American women, a higher *APOE*4 dosage (from 0 to 2) was linked to slower decline on a test of attention (BTA: γ_12_ = + 0.106 ± 0.035, *p* = 0.002), while no such association was detected among African American men with respect to APOE4 dosage and change in BTA over time. *APOE*2 and *APOE*4 dosages showed inconsistent results in other domains of cognition overall and across racial groups that did not survive correction for multiple testing.

### Previous studies

Prior work evaluating associations between *APOE* genotype and cognitive health has also reported heterogeneity in the magnitude of these associations across racial/ethnic groups and depending on the phenotype under study. For example, several studies [1–4, 15] have reported larger associations between the ε4 allele and incident AD dementia among European Americans compared to their African American counterparts yet relatively little work has examined variation in cognitive decline—as opposed to incident dementia—among European and African American carriers of the ε4 allele. In a recent study by Rajan and colleagues [43], the authors examined disparities in the association between the ε4 allele and cognitive decline among European and African American men and women using a composite measure of cognitive function that included assessments of episodic memory, perceptual speed, executive function, general orientation, and global cognition. The authors found that, in addition to being more likely to have at least one ε4 allele, African American adults also had lower cognitive function at baseline in addition to accelerated cognitive decline over nearly 10 years of follow-up compared to European Americans in the sample [43]. Moreover, carriers of the ε4 allele—irrespective of ancestry—were more likely to exhibit rapid and moderate cognitive decline relative to slow decline [43]. This finding differs from ours, as we mainly found baseline differences in cognitive performance (better performance among Whites compared with African Americans) in most tests, with only one test showing differences in terms of decline (BVRT), whereby Whites had slower decline than African Americans. In addition, our finding of a direct association between the ε4 allele dosage and decline on a test of verbal memory was limited to Whites, unlike the study by Rajan and colleagues [43]. In the latter study [43], genotypic frequencies and differences by race were comparable to our sample, with African Americans’ vs. Whites’ frequencies for each genotype being: APOE ε2/ε3 (14% vs 12%), ε2/ε4 (4% vs 2%), ε3/ε4 (29% vs 22%), and ε4/ε4 (4% vs 2%) genotypes. Similar to our study, an addition of 1 e4 allele was associated with an annual faster decline on global cognition by ~ 0.05 SD, or an equivalent difference of 0.50 SD over a period of 10 years. While Rajan and colleagues found this effect size for both Whites and African Americans, our study found this effect size only among Whites, and for verbal memory rather than global mental status [43]. Unlike Rajan et al., we did not detect associations with APOE2 dosages, possibly due to the lower genotypic frequencies with ε2 alleles compared to their study or lower cognitive performance variability, thus reducing statistical power. Our study also included other potentially confounding covariates, such as a measure of literacy, which was not accounted for in the models for most past studies that tested associations between APOE genotypes and cognitive decline.

In response to the consistent finding that the ε4 allele may be detrimental to cognitive health according to previous studies (e.g., [43]), investigators have turned towards understanding resilience factors against cognitive decline among carriers of the ε4 allele. For example, in a study by Kaup and colleagues [44], the authors tested factors that predicted cognitive resilience among carriers of the ε4 allele. The authors reported variation with respect to the factors identified among white and black adults and their associated magnitudes which may be suggestive of gene-environment interactions in the associations between race, *APOE*, and cognitive decline. Our main findings were not affected by introducing lifestyle or health-related factors into the models. Other secondary findings that did not survive correction for multiple testing were affected by inclusion of factors such as smoking and diet quality. Future studies should uncover how these lifestyle and health-related factors play a role in the association between APOE and various neuro-cognitive outcomes.

Sex differences in the association between *APOE* genotypes or allele dosages with various cognitive outcomes were mostly studied among individuals of European ancestry [5, 6]. In one study by Beydoun et al., it was found that even though *APOE*4(+) status (i.e., any ε4 allele) predicted dementia significantly (hazard ratio [HR] = 2.89; 95% confidence interval [CI], 1.93–4.33), with nonsignificant sex differences, women had significantly stronger positive associations than men between *APOE*4(+) status and impairment or decline on the California Verbal Learning Test (CVLT; delayed recall and List A total recall) and on Verbal Fluency Test-Categories [5]. In another study, female *APOE*4 carriers have faster rates of memory decline than their male counterparts among MCI individuals [6]. Our present study did not replicate this finding in terms of *APOE*4 dosage’s association with cognitive decline (e.g., in verbal memory or fluency) being stronger among White women *vs*. White men, and sex differences as such were not detected in the total sample. Such lack of heterogeneity by sex was found in at least one other comparable prospective cohort study [22].

### Biological mechanisms

In terms of biological plausibility, *APOE* genotype has long been associated with cardiovascular disease, dementia, and Alzheimer’s disease [45]. More recently, researchers have reported associations between *APOE* genotype and neurobiological markers of aging at various stages of the life course [46]. The three alleles that comprise *APOE* vary with respect to their affinity for binding to serum cholesterol which, in turn, influences the extent to which individuals can metabolize dietary fat in the blood [47]. Individual carriers of the ε2 allele have lower total serum cholesterol compared to ε3/ε3, on average, whereas carriers of ε4 tend to have higher levels relative to *APOE*3 homozygotes [47]; an association that has been reported at younger ages as well [45]. Nevertheless, it has been also shown that, unlike ε2/ε3, the ε2/ε2 *APOE* genotype was linked to higher levels of serum triglycerides which may exacerbate cerebrovascular disease, when compared with ε3/ε3 [48]. In fact, recent evidence points to a strong relationship between serum triglycerides and several markers of neurodegeneration [49]. This may explain in part the adverse (though marginal) link found in our study between *APOE*2 allelic dosage and decline on global mental status. However, an in-depth analysis of the association between *APOE* genotypes with serum lipid trajectories over time may be needed, which in turn can be studied in relation to markers of neurodegeneration. The putative protective effect of *APOE*4 dosage among African American women on performance in the domain of attention over time requires replication and deserves further study with respect to underlying mechanisms. Specifically, the difference between African American men and women in this association may be the result of biological or hormonal sex differences, as it could be caused by socioenvironmental factors related to gender. Finally, a recent study by Morris et al. found that lower CSF concentrations of total tau and phosphorylated tau181 in African American individuals may reflect a significant race by *APOE*4 dosage interaction, suggesting a differential effect of this Alzheimer risk variant in African American individuals compared with white individuals [50]. Nevertheless, this study included a limited number of APOE4 carriers among African Americans in that sample [50]. Despite the lack of assessment for ethnicity in our study, our findings may have been affected if sufficient number of participants had a Latino ethnicity as well. However, Latinos is a heterogeneous ethnicity, genetically, biologically, and culturally, and thus, it is difficult to speculate as to the direction of the change in our key findings.

### Strengths and limitations

The strengths of our study include its large sample size allowing us to detect small effects, both cross-sectional and longitudinal, within the context of mixed-effects linear regression models with outcomes being 11 test scores spanning various domains of cognition, in a socioeconomically and racially diverse sample of middle-aged urban adults. Given the possibility of small effect being detected, some of the main findings were presented in terms of effect size, using standardized regression coefficients, in addition to showing their statistical significance. The larger sample size and adequate balance by race and sex also allowed testing for effect modification by both of these socio-demographic factors. Our analysis adjusted for important potential confounders, corrected for multiple testing, adjusted for selection bias due to missingness in outcome data using a 2-stage Heckman selection model, included multiple imputation of covariates, and formally tested effect modification by race and sex. Our results can be extrapolated to many African American and White middle-aged urban adult populations, as HANDLS is representative of 14 urban settings across the USA [35]. Despite these strengths, our study has several limitations. Those include the relatively small number of ε2 and ε4 carriers after models were stratified by race and by race × sex, particularly for ε2 alleles (< 20 participants overall, < 10 for each race group). Nevertheless, our analyses included *APOE*2 or *APOE*4 dosages as an ordinal variable (0,1,2) as opposed to studying genotypes using a common referent approach (e.g., 1 vs. 0; 2 vs. 0). This improved statistical power in our models, although it is worth noting that for some race × sex analyses, the APOE dosage may be representing a move from “0” to “1” dosage only, given the lack of availability for the “2” category. Thus, our main focus was on the stratification by race only.

### Conclusions

In conclusion, we found putative adverse associations between the *APOE* ε4 allele dosage and cognitive decline in the memory domain among Whites, while among African American women, *APOE* ε4 allele dosage had a potential protective effect on the domain of attention over time. Such domain-specific inconsistencies have been reported in other studies, particularly when comparing the effect of *APOE*4 allele status or dosage on memory to that on other more crystallized domains of cognition (e.g., verbal fluency or attention). *APOE*2 dosage appeared to have less consistent associations with various domains of cognition across racial groups. Further longitudinal studies are needed to replicate our race and sex-specific findings.

## Supplementary Information

**Additional file 1.**

**Additional file 2:** **Figure S1.** Standard deviations (SD) of cognitive performance test scores over period of follow-up for each of 11 cognitive tests, in eligible sample (*N* = 1,770, k = 1.7 observations/participant), HANDLS 2004-2013^a^. Abbreviations*:* AF = Animal Fluency; BTA = Brief Test of Attention; BVRT = Benton Visual Retention Test; CDT = Clock Drawing Test; CVLT-DFR = California Verbal Learning Test-Delayed Free Recall; CVLT-List A = California Verbal Learning Test-List A; DS-B=Digits Span-Backward; DS-F=Digits Span-Forward; HANDLS = Healthy Aging in Neighborhood of Diversity across the Lifespan; MMSE = Mini-Mental State Examination; SD=Standard Deviation; TRAILS A = Trailmaking Test, Part A; TRAILS B = Trailmaking Test, part B. ^a^ SD computed for each of 11 cognitive test performances over v1 and/or v2 for eligible sample (*N* = 1,770, k = 1.7 observations/participant). The SD is used to contextualize the effects obtained in mixed-effects linear regression models as a proportion of the SD.

## Data Availability

The study protocol (09-AG-N248) received approval from the National Institute on Environmental Health Sciences’ Institutional Review Board (IRB) of the National Institutes of Health (NIH). Upon request, data can be made available to researchers with approved proposals, after they have agreed to confidentiality as required by our IRB. Policies are publicized on https://handls.nih.gov. Data access request can be sent to principal investigators (PI) or the study manager, Jennifer Norbeck at norbeckje@mail.nih.gov. These data are owned by the National Institute on Aging at the NIH. The PIs have made those data restricted to the public for two main reasons: “(1) The study collects medical, psychological, cognitive, and psychosocial information on racial and poverty differences that could be misconstrued or willfully manipulated to promote racial discrimination; and (2) Although the sample is fairly large, there are sufficient identifiers that the PIs cannot guarantee absolute confidentiality for every participant as we have stated in acquiring our confidentiality certificate” [51].

## Abbreviations

AF: Animal fluency test
APOE: Apolipoprotein E
APOE2: Apolipoprotein ε2
APOE4: Apolipoprotein ε4
BVRT: Benton Visual Retention Test
BMI: Body mass index
BTA: Brief Test of Attention
CVLT-DFR: California Verbal Learning Test–Free Recall Long Delay
CVLT-List-A: California Verbal Learning Test–List A
CVD: Cardiovascular disease
CES-D: Center for Epidemiological Studies-Depression scale
CDT: Clock Draw Test
CI: Confidence interval
DS-B: Digit Span-Backward Test
DS-F: Digit Span-Forward Test
HEI-2010: Healthy Eating Index 2010
HANDLS: Healthy Aging in Neighborhoods of Diversity across the Life Span
HS: High school
MRV: Medical research vehicles
MMSE: Mini-Mental State Examination
NDI: National Death Index
SD: Standard deviation
SE: Standard error
Trails A: Trailmaking Test A
Trails B: Trailmaking Test B
WRAT-3: Wide Range Achievement Test, third edition
