# Supplementary material for: Race, APOE genotypes, and cognitive decline among middle-aged urban adults
Source: Alzheimers Res Ther. 2021 Jun 30;13:120. doi: 10.1186/s13195-021-00855-y (PMC8247163; doi:10.1186/s13195-021-00855-y)
Supplement: Supplementary file 1 — Additional file 1. [file 13195_2021_855_MOESM1_ESM.docx]

**Method S1: Description of cognitive tests, literacy and the CES-D**

*Mini-Mental State Examination (MMSE)*

The MMSE ^1^ is a cognitive screener that captures global cognitive functioning by briefly measuring orientation, concentration, immediate and short-term memory, language and constructional praxis. Scores range from 0 to 30. Higher scores suggest better cognitive function.

*California Verbal Learning Test (CVLT)*

The CVLT ^2^ is a verbal learning and memory test that includes a 16-item word list. A modified version of the CVLT was used with three, as opposed to five, learning trials. Cued recall was not administered. To capture verbal learning and memory, CVLT outcomes variables were total correct score for List A (learning) and List A long-delay free recall (memory). The learning score ranged from 0 to 48 and the memory score ranged from 0 to 16. Higher scores indicate better verbal learning and memory. A more comprehensive description of CVLT can be found elsewhere ^2^.

*Benton Visual Retention Test* (BVRT)

The BVRT ^3^ is a measure of nonverbal memory and visuo-constructional abilities. Administration A, Form D was used. A modified error scoring system based off the BVRT manual was used to guide two trained examiners in scoring the BVRT. Resolution of discrepancies in scoring were attempted by the two examiners, however, if a consensus could not be achieved, MKT, a research psychologist, provided the score. The outcome variable was total errors, with higher values indicating lower visual memory scores.

*Brief Test of Attention (BTA)*

For the BTA ^4^, a test of divided auditory attention, the examiner administered up to 10 trials of letters and numbers (4-18 items) that increased in length with each trial. Only the numbers portion of the test was administered. For each trial, participants were asked to disregard the number of letters read, while tracking how many numbers were recited. They were also told to keep their hands in fists to avoid finger counting. The outcome variable was the total number of correct trials.

*Animal Fluency (AF)*

Category fluency^5, 6^ is a measure of semantic verbal fluency, where participants are asked to generate as many animals as possible within a 60 second duration. Higher scores indicate better category fluency. The outcome variable was the total number of correctly generated words (i.e., words that were *not* intrusions and perseverations).

*Digit Span Forward and Backward (DS-F and DS-B)*

The Wechsler Adult Intelligence Scale, Revised ^7^ Digit Span Forward and Backward primarily capture attention and working memory, a component of executive function. The tests were administered according to the manual’s instructions. The outcome variable was the total score, which was the total number of correct answers for each test.

*Clock Drawing Test – Clock to Command (CDT)*

The Clock Drawing Test ^8^ is a measure of visuo-spatial abilities, that also captures elements of memory and executive function. Participants are instructed to draw a clock, put in all of the numbers, and set the hands to 10 minutes past 11. Performance is based off correct drawings of the clock face (0-2), numbers (0-4) and hands (0-4). Scores ranged from 0 to 10, with higher scores indicating better performance. Participants who did not score a perfect score on the command portion of the test were also asked to copy a clock with the hands set to 10 minutes after 11.

*Trail Making Tests A and B (TRAILS A and B)*

The Trail Making Tests A and B^9^ primarily capture attention and executive functioning, respectively. The main executive function subdomain that TRAILS B captures is set-shifting and cognitive control. Both trials also measure visuo-motor scanning and processing speed. Participants were asked to draw a line between consecutive numbers (TRAILS A) and alternate between numbers and letters (TRAILS B) as quickly as they could. They were informed that they were being timed. The examiner pointed out errors that were then corrected by the participant. Errors were captured via increased time. Scores for TRAILS A and B reflected seconds to completion, where higher scores indicate poorer performance.

*Wide Range Achievement Test – 3^rd^ Edition: Word and Letter Reading Subtest (WRAT)*

The WRAT Word and Letter Reading Subtest ^10^ is a test of reading ability that is often used as a proxy for literacy and quality of education. Participants were instructed to correctly read a list of 50 words that increased in difficulty. If the first five words were not correctly pronounced, letter reading was also administered. Standard instructions were used with the tan form. The outcome variable used was the total number of correctly pronounced words.

*Center for Epidemiological Studies Depression Scale (CES-D)*

The CES-D ^11^ is a 20-item measure of depressive symptomatology. Participants are asked to consider the frequency and severity of their symptoms over the last week. Scores ranged from 0 to 60. Scores of >16 indicated significant depressive symptoms and scores of >20 indicated a clinically significant amount of depressive symptoms.

**Method S2**: Mixed-effects regression models

The main multiple mixed-effects regression models can be summarized as follows:

**Multi-level models** vs. **Composite models**

| **Eq.**  **1.1-1.4** |  |  |  |
| --- | --- | --- | --- |

Where Y_ij_ is the outcome (Each cognitive test score measured at v_1_ and/or v_2_) for each individual “i” and visit “j”; is the level-1 intercept for individual i; is the level-1 slope for individual i; is the level-2 intercept of the random intercept ; is the level-2 intercept of the slope ; is a vector of fixed covariates for each individual *i* that are used to predict level-1 intercepts and slopes, which can include socio-demographic variables among others. In this analysis, mixed-effects regression models included alternate exposures (X_ij_), namely APOE2/4 allelic dosages, and specific sets of covariates (Z_ij_). However, separate models that did not include (X_ij_) or (Z_ij_) were used for the sole purpose of predicting empirical bayes estimators for change in cognitive performance for each test, with *TIME* as the only variable in the multi-level model. and are level-2 disturbances; is the within-person level-1 disturbance ^12^.

It is worth noting that the models were fit using the entire HANDLS cohort with complete data on either v1 or v2 on cognitive tests was used to improve reliability of predicted estimates.

**Supplemental References**

1. Folstein MF, Folstein SE, McHugh PR. "Mini-mental state". A practical method for grading the cognitive state of patients for the clinician. *J Psychiatr Res* 1975; **12**(3)**:** 189-198.

2. Delis DC, Freeland J, Kramer JH, Kaplan E. Integrating clinical assessment with cognitive neuroscience: construct validation of the California Verbal Learning Test. *J Consult Clin Psychol* 1988; **56**(1)**:** 123-130.

3. Benton AL (ed). *Revised visual retention test (fifth edition)*. The Psychological Corportation: New York, 1974.

4. Schretlen D, Bobholz JH, Brandt J. Development and psychometric properties of the Brief Test of Attention. *Clinical Neuropsychologist* 1996; **10**(1)**:** 80-89.

5. Morris JC, Heyman A, Mohs RC, Hughes JP, van Belle G, Fillenbaum G *et al.* The Consortium to Establish a Registry for Alzheimer's Disease (CERAD). Part I. Clinical and neuropsychological assessment of Alzheimer's disease. *Neurology* 1989; **39**(9)**:** 1159-1165.

6. Morris JC, Mohs RC, Rogers H, Fillenbaum G, Heyman A. Consortium to establish a registry for Alzheimer's disease (CERAD) clinical and neuropsychological assessment of Alzheimer's disease. *Psychopharmacol Bull* 1988; **24**(4)**:** 641-652.

7. Wechsler D. WAIS-R manual. Cleveland: The Psychological Corporation; 1981.

8. Rouleau I, Salmon DP, Butters N, Kennedy C, McGuire K. QUANTITATIVE AND QUALITATIVE ANALYSES OF CLOCK DRAWINGS IN ALZHEIMERS AND HUNTINGTONS-DISEASE. *Brain and Cognition* 1992; **18**(1)**:** 70-87.

9. Reitan R. *Trail Making Test: Manual for Administration and Scoring*. Reitan Neuropsychological Laboratory: Tucson, AZ, 1992.

10. Wilkinson GS. *Wide Range Achievement Test–Revision 3*. Jastak Association: Wilmington, DE, 1993.

11. Nguyen HT, Kitner-Triolo M, Evans MK, Zonderman AB. Factorial invariance of the CES-D in low socioeconomic status African Americans compared with a nationally representative sample. *Psychiatry research* 2004; **126**(2)**:** 177-187.

12. Blackwell E, de Leon CF, Miller GE. Applying mixed regression models to the analysis of repeated-measures data in psychosomatic medicine. *Psychosom Med* 2006; **68**(6)**:** 870-878.

**TABLE S1**. *APOE*2 and *APOE*4 allelic dosages and their association with cognitive performance at v1 and change over time: sex-specific mixed-effects linear regression models: HANDLS 2004-2013^a^

|  |  | *APOE2* allelic dosage | | *APOE4* allelic dosage | |  |
| --- | --- | --- | --- | --- | --- | --- |
|  |  | Model 1 | Model 2 | Model 1 | Model 2 |  |
|  |  | *γ*±SE | *γ*±SE | *γ*±SE | *γ*±SE |  |
| **Women** |  | **(N=1,012, k=1.8)** | **(N=1,012, k=1.8)** | **(N=1,012, k=1.8)** | **(N=1,012, k=1.8)** |  |
| *Outcome=Cognitive performance test score* |  |  |  |  |  |  |
| Normalized MMSE |  |  |  |  |  |  |
| Exposure, *γ_0a_* |  | -0.0692±1.0328 | -0.1035±0.9085 | -0.5406±0.7787 | -0.2203±0.6847 |  |
| Exposure×TIME, *γ_1a_* |  | -0.4488±0.2517 | -0.4276±0.2487 | +0.3420±0.1934 | +0.2799±0.1910 |  |
| CVLT-List A |  |  |  |  |  |  |
| Exposure, *γ_0a_* |  | -0.1250±0.5081 | -0.1751±0.4738 | +0.1882±0.3864 | +0.2349±0.3595 |  |
| Exposure×TIME, *γ_1a_* |  | +0.0812±0.1361 | +0.0681±0.1351 | -0.0929±0.1050 | -0.1109±0.1042 |  |
| CVLT-DFR |  |  |  |  |  |  |
| Exposure, *γ_0a_* |  | -0.1235±0.2358 | -0.1472±0.2219 | -0.0303±0.1792 | -0.0199±0.1685 |  |
| Exposure×TIME, *γ_1a_* |  | +0.0609±0.0539 | +0.0600±0.0535 | -0.0434±0.0419 | -0.0411±0.0415 |  |
| BVRT |  |  |  |  |  |  |
| Exposure, *γ_0a_* |  | -0.3205±0.3543 | -0.2024±0.3358 | +0.5477±0.2662 | +0.4921±0.2522 |  |
| Exposure×TIME, *γ_1a_* |  | +0.1142±0.0830 | +0.0883±0.0833 | -0.0617±0.0639 | -0.0529±0.0640 |  |
| BTA |  |  |  |  |  |  |
| Exposure, *γ_0a_* |  | +0.0098±0.1640 | -0.0804±0.1564 | -0.1193±0.1232 | -0.0531±0.1175 |  |
| Exposure×TIME, *γ_1a_* |  | +0.0065±0.0380 | +0.0245±0.0377 | +0.0737±0.0292 | +0.0668±0.0289 |  |
| AF |  |  |  |  |  |  |
| Exposure, *γ_0a_* |  | +0.2353±0.3825 | +0.2081±0.3622 | +0.0315±0.2883 | +0.0569±0.2723 |  |
| Exposure×TIME, *γ_1a_* |  | -0.0001±0.0716 | +0.0084±0.0723 | +0.0098±0.0553^c^ | +0.0039±0.0557 |  |
| DS-F |  |  |  |  |  |  |
| Exposure, *γ_0a_* |  | +0.0377±0.1574 | +0.0067±0.1444 | -0.1110±0.1186 | -0.0769±0.1088 |  |
| Exposure×TIME, *γ_1a_* |  | -0.0074±0.0317 | +0.0009±0.0318 | +0.0417±0.0240 | +0.0455±0.0242 |  |
| DS-B |  |  |  |  |  |  |
| Exposure, *γ_0a_* |  | -0.2107±0.1557 | -0.2352±0.1383 | +0.0695±0.1175 | +0.1179±0.1043 |  |
| Exposure×TIME, *γ_1a_* |  | +0.0237±0.0316 | +0.0301±0.0314 | +0.0122±0.0242 | +0.0054±0.0241 |  |
| CDT |  |  |  |  |  |  |
| Exposure, *γ_0a_* |  | +0.0931±0.0862 | +0.0776±0.0863 | +0.0264±0.0667 | +0.0329±0.065 |  |
| Exposure×TIME, *γ_1a_* |  | -0.0313±0.0232 | -0.0319±0.0233 | -0.036±0.0179^c^ | -0.0376±0.0180 |  |
| TRAILS A |  |  |  |  |  |  |
| Exposure, *γ_0a_* |  | -0.2813±2.6157 | +0.0357±2.6039 | +0.3004±1.9654 | +0.0165±1.9555 |  |
| Exposure×TIME, *γ_1a_* |  | +0.2833±0.7560 | +0.2816±0.7558 | -0.0896±0.5835 | -0.1278±0.5821 |  |
| TRAILS B |  |  |  |  |  |  |
| Exposure, *γ_0a_* |  | +4.1862±10.7782 | +8.8677±10.3559 | +6.7943±8.1019 | +4.2992±7.7547 |  |
| Exposure×TIME, *γ_1a_* |  | -1.4360±2.7072 | -2.7157±2.7251 | -0.9363±2.0833 | -0.5230±2.0861 |  |
| **Men** |  | **(N=758, k=1.7)** | **(N=758, k=1.7)** | **(N=758, k=1.7)** | **(N=758, k=1.7)** |  |
| *Outcome=Cognitive performance test score* |  |  |  |  |  |  |
| MMSE |  |  |  |  |  |  |
| Exposure, *γ_0a_* |  | -1.5206±1.3089 | -0.4705±1.0802 | +0.7597±0.9933 | +0.0862±0.8238 |  |
| Exposure×TIME, *γ_1a_* |  | -0.3303±0.3348 | -0.4132±0.3188 | +0.0007±0.2458 | +0.0717±0.2375 |  |
| CVLT-List A |  |  |  |  |  |  |
| Exposure, *γ_0a_* |  | -0.4673±0.5328 | -0.1014±0.4906 | +0.1662±0.4103 | +0.0798±0.3797 |  |
| Exposure×TIME, *γ_1a_* |  | +0.1092±0.1464 | +0.0562±0.1448 | -0.1438±0.1083 | -0.1401±0.1091 |  |
| CVLT-DFR |  |  |  |  |  |  |
| Exposure, *γ_0a_* |  | -0.294±0.2518 | -0.1293±0.2393 | +0.0510±0.1943 | +0.0391±0.1829 |  |
| Exposure×TIME, *γ_1a_* |  | +0.1214±0.0648 | +0.0902±0.0767 | -0.0050±0.0485 | -0.0042±0.0488 |  |
| BVRT |  |  |  |  |  |  |
| Exposure, *γ_0a_* |  | +0.0253±0.4143 | -0.223±0.3727 | +0.0983±0.3135 | +0.1116±0.2838 |  |
| Exposure×TIME, *γ_1a_* |  | -0.0142±0.0947 | +0.0204±0.0937 | +0.0322±0.0695 | +0.0274±0.0697 |  |
| BTA |  |  |  |  |  |  |
| Exposure, *γ_0a_* |  | -0.0108±0.1824 | +0.1214±0.1715 | -0.0573±0.1377 | -0.0975±0.1298 |  |
| Exposure×TIME, *γ_1a_* |  | +0.0156±0.0457 | -0.0054±0.0459 | -0.0297±0.0341 | -0.0234±0.0346 |  |
| AF |  |  |  |  |  |  |
| Exposure, *γ_0a_* |  | -0.3457±0.4513 | -0.1172±0.4268 | +0.2054±0.3424 | +0.1271±0.3250 |  |
| Exposure×TIME, *γ_1a_* |  | -0.1547±0.0943 | -0.1653±0.0950 | -0.0150±0.0696^c^ | -0.0268±0.0710 |  |
| DS-F |  |  |  |  |  |  |
| Exposure, *γ_0a_* |  | -0.1320±0.1909 | -0.0267±0.1715 | +0.1248±0.1444 | +0.0590±0.1305 |  |
| Exposure×TIME, *γ_1a_* |  | +0.0223±0.0377 | +0.0146±0.0376 | +0.0335±0.0278 | +0.0336±0.0281 |  |
| DS-B |  |  |  |  |  |  |
| Exposure, *γ_0a_* |  | +0.0559±0.1847 | +0.1529±0.1624 | +0.0269±0.1408 | -0.0387±0.1249 |  |
| Exposure×TIME, *γ_1a_* |  | -0.0371±0.0382 | -0.0462±0.0381 | -0.0073±0.0287 | -0.0121±0.0290 |  |
| CDT |  |  |  |  |  |  |
| Exposure, *γ_0a_* |  | -0.0683±0.1023 | -0.0476±0.0997 | -0.1144±0.0776 | -0.1224±0.0760 |  |
| Exposure×TIME, *γ_1a_* |  | +0.0195±0.0282 | +0.0196±0.0280 | +0.0243±0.0209^c^ | +0.0297±0.0210 |  |
| TRAILS A |  |  |  |  |  |  |
| Exposure, *γ_0a_* |  | -4.1235±3.6420 | -4.115±3.5999 | +1.0565±2.7675 | +1.1531±2.7451 |  |
| Exposure×TIME, *γ_1a_* |  | +1.3003±1.3346 | +1.4649±1.3402 | -0.3215±0.9857 | -0.5483±1.0005 |  |
| TRAILS B |  |  |  |  |  |  |
| Exposure, *γ_0a_* |  | +1.1939±12.8916 | -8.3297±11.4572 | -2.8115±9.7645 | +1.8260±8.7188 |  |
| Exposure×TIME, *γ_1a_* |  | -1.4531±2.5937 | -0.4554±2.6353 | +1.1800±1.9045 | +0.8587±1.9511 |  |

Abbreviations*:* AF=Animal Fluency; *APOE*=Apolipoprotein E genotype; BMI=Body Mass Index; BTA=Brief Test of Attention; BVRT=Benton Visual Retention Test; CDT=Clock Drawing Test; CES-D=Center for Epidemiologic Studies-Depression; CVLT-DFR=California Verbal Learning Test-Delayed Free Recall; CVLT-List A=California Verbal Learning Test-List A; DS-B=Digits Span-Backward; DS-F=Digits Span-Forward; HANDLS = Healthy Aging in Neighborhood of Diversity across the Lifespan; HEI-2010=Healthy Eating Index, 2010 version; HS = High school; MMSE=Mini-Mental State Examination; SD=Standard Deviation; TRAILS A=Trailmaking Test, Part A; TRAILS B=Trailmaking Test, part B; WRAT-3 = Wide Range Achievement Test, 3rd revision; X = mean.

^a^ Models 1A.1-1K. included each of *APOE2* or *APOE4* allelic dosages, separately as the main predictor for v1 cognitive performance and cognitive change over time (11 test scores), using a series of mixed-effects linear regression models, carried out in the overall population and stratified by race. These models adjusted only for age, sex, race, poverty status, and the inverse mills ratio. Models 2A.1-2K. followed a similar approach but adjusted further for selected socio-demographic, lifestyle and health-related factors, namely educational attainment, the WRAT-3 score, current drug use, current tobacco use, body mass index, self-rated health, co-morbidity index, HEI-2010, total energy intake, and the CES-D total score.

^b^ p<0.05 for Sex×(*APOE*2 or *APOE*4) in models that are unstratified by race to which this 2-way interaction was included.

^c^ p<0.05 for Sex×(*APOE*2 or *APOE*4)×*TIME* in models that are unstratified by race to which this 3-way interaction was included.

**p* < 0.05** *p* < 0.01; *** *p* < 0.001, test for null hypothesis of γ=0. Bolded values passed correction for multiple testing.

**TABLE S2**. *APOE*2 and *APOE*4 allelic dosages and their association with cognitive performance at v1 and change over time: sex-specific mixed-effects linear regression models: HANDLS 2004-2013^a^

|  |  |  | |  |
| --- | --- | --- | --- | --- |
|  |  | *APOE2* allelic dosage | *APOE4* allelic dosage | |
|  |  | *γ*±SE | *γ*±SE |  |
| **White women** |  | **(N=447, k=1.8)** | **(N=447, k=1.8)** |  |
| *Outcome=Cognitive performance test score* |  |  |  |  |
| Normalized MMSE |  |  |  |  |
| Exposure, *γ_0a_* |  | +1.5029±1.5079 | -0.7103±1.1241 |  |
| Exposure×TIME, *γ_1a_* |  | -0.9175±0.4476* | +0.7673±0.3265* |  |
| CVLT-List A |  |  |  |  |
| Exposure, *γ_0a_* |  | +1.2396±0.8984 | +0.0921±0.6742 |  |
| Exposure×TIME, *γ_1a_* |  | +0.1048±0.2748 | -0.3765±0.1985 |  |
| CVLT-DFR |  |  |  |  |
| Exposure, *γ_0a_* |  | +0.1703±0.4088 | -0.1328±0.3059 |  |
| Exposure×TIME, *γ_1a_* |  | +0.1413±0.1098 | -0.1076±0.0794 |  |
| BVRT |  |  |  |  |
| Exposure, *γ_0a_* |  | +0.1916±0.5248 | +0.5035±0.390 |  |
| Exposure×TIME, *γ_1a_* |  | -0.0049±0.1313 | +0.0215±0.0945 |  |
| BTA |  |  |  |  |
| Exposure, *γ_0a_* |  | -0.3549±0.2607 | +0.0514±0.1944 |  |
| Exposure×TIME, *γ_1a_* |  | +0.1591±0.0685* | -0.0258±0.0507 |  |
| AF |  |  |  |  |
| Exposure, *γ_0a_* |  | +1.0456±0.6499 | +0.2903±0.4830 |  |
| Exposure×TIME, *γ_1a_* |  | +0.0245±0.1461 | +0.0179±0.1060 |  |
| DS-F |  |  |  |  |
| Exposure, *γ_0a_* |  | -0.1439±0.2444 | -0.2698±0.1816 |  |
| Exposure×TIME, *γ_1a_* |  | +0.0383±0.0642 | +0.0620±0.0473 |  |
| DS-B |  |  |  |  |
| Exposure, *γ_0a_* |  | -0.2799±0.2484 | +0.0843±0.1859 |  |
| Exposure×TIME, *γ_1a_* |  | +0.0304±0.0579 | +0.0025±0.0427 |  |
| CDT |  |  |  |  |
| Exposure, *γ_0a_* |  | -0.0499±0.1410 | -0.0294±0.1055 |  |
| Exposure×TIME, *γ_1a_* |  | -0.0322±0.0410 | -0.0427±0.0300 |  |
| TRAILS A |  |  |  |  |
| Exposure, *γ_0a_* |  | -0.7286±1.3001 | +0.9429±0.9679 |  |
| Exposure×TIME, *γ_1a_* |  | -0.7334±0.3562* | +0.5896±0.2586* |  |
| TRAILS B |  |  |  |  |
| Exposure, *γ_0a_* |  | +14.9270±14.4038 | +13.4108±10.7664 |  |
| Exposure×TIME, *γ_1a_* |  | -2.6876±3.9499 | -2.8044±2.8754 |  |
| **White men** |  | **(N=347, k=1.7)** | **(N=347, k=1.7)** |  |
| *Outcome=Cognitive performance test score* |  |  |  |  |
| MMSE |  |  |  |  |
| Exposure, *γ_0a_* |  | -2.7608±1.6377 | +1.5625±1.2856 |  |
| Exposure×TIME, *γ_1a_* |  | +0.3896±0.5430 | -0.1277±0.3995 |  |
| CVLT-List A |  |  |  |  |
| Exposure, *γ_0a_* |  | -0.4199±0.7457 | +0.8338±0.5870 |  |
| Exposure×TIME, *γ_1a_* |  | +0.0439±0.2512 | -0.3435±0.1847 |  |
| CVLT-DFR |  |  |  |  |
| Exposure, *γ_0a_* |  | -0.1400±0.3623 | +0.3281±0.2828 |  |
| Exposure×TIME, *γ_1a_* |  | +0.1412±0.1047 | -0.1128±0.0765 |  |
| BVRT |  |  |  |  |
| Exposure, *γ_0a_* |  | +0.0206±0.4977 | +0.1179±0.3903 |  |
| Exposure×TIME, *γ_1a_* |  | +0.2025±0.1440 | +0.0289±0.1061 |  |
| BTA |  |  |  |  |
| Exposure, *γ_0a_* |  | +0.2281±0.2607 | -0.1481±0.1948 |  |
| Exposure×TIME, *γ_1a_* |  | -0.1119±0.0724 | -0.0562±0.0524 |  |
| AF |  |  |  |  |
| Exposure, *γ_0a_* |  | -0.1687±0.6440 | +0.3381±0.5020 |  |
| Exposure×TIME, *γ_1a_* |  | -0.1628±0.1665 | -0.0564±0.1227 |  |
| DS-F |  |  |  |  |
| Exposure, *γ_0a_* |  | -0.0628±0.2660 | -0.0925±0.2068 |  |
| Exposure×TIME, *γ_1a_* |  | -0.0412±0.0649 | +0.0323±0.0476 |  |
| DS-B |  |  |  |  |
| Exposure, *γ_0a_* |  | -0.0520±0.2633 | -0.0028±0.2082 |  |
| Exposure×TIME, *γ_1a_* |  | -0.0134±0.0640 | +0.0289±0.0492 |  |
| CDT |  |  |  |  |
| Exposure, *γ_0a_* |  | -0.0635±0.1541 | -0.0008±0.1211 |  |
| Exposure×TIME, *γ_1a_* |  | -0.0562±0.0477 | -0.0243±0.0356 |  |
| TRAILS A |  |  |  |  |
| Exposure, *γ_0a_* |  | -1.2362±4.5465 | +3.6829±3.5566 |  |
| Exposure×TIME, *γ_1a_* |  | +0.9123±1.5922 | -1.5548±1.1957 |  |
| TRAILS B |  |  |  |  |
| Exposure, *γ_0a_* |  | +0.3538±14.7783 | +25.464±11.4012* |  |
| Exposure×TIME, *γ_1a_* |  | -4.7475±3.2663 | -4.5536±2.4883 |  |
| **African American women** |  | **(N=565, k=1.8)** | **(N=565, k=1.8)** |  |
| *Outcome=Cognitive performance test score* |  |  |  |  |
| MMSE |  |  |  |  |
| Exposure, *γ_0a_* |  | -0.9872±1.1395 | +0.1643±0.8698 |  |
| Exposure×TIME, *γ_1a_* |  | -0.2479±0.3043 | +0.0696±0.2380 |  |
| CVLT-List A |  |  |  |  |
| Exposure, *γ_0a_* |  | -0.8685±0.5315 | +0.448±0.4084 |  |
| Exposure×TIME, *γ_1a_* |  | +0.0708±0.1511 | -0.0101±0.1184 |  |
| CVLT-DFR |  |  |  |  |
| Exposure, *γ_0a_* |  | -0.4052±0.2552 | +0.0953±0.1955 |  |
| Exposure×TIME, *γ_1a_* |  | +0.0615±0.0594 | -0.0185±0.0465 |  |
| BVRT |  |  |  |  |
| Exposure, *γ_0a_* |  | -0.3755±0.4373 | +0.4324±0.3331 |  |
| Exposure×TIME, *γ_1a_* |  | +0.1300±0.1094 | -0.0796±0.0862 |  |
| BTA |  |  |  |  |
| Exposure, *γ_0a_* |  | +0.0174±0.1945 | -0.0930±0.1480 |  |
| Exposure×TIME, *γ_1a_* |  | -0.0314±0.0447 | **+0.1077±0.0346**** |  |
| AF |  |  |  |  |
| Exposure, *γ_0a_* |  | -0.3265±0.4162 | -0.0118±0.3170 |  |
| Exposure×TIME, *γ_1a_* |  | +0.0030±0.0818 | -0.0069±0.0643 |  |
| DS-F |  |  |  |  |
| Exposure, *γ_0a_* |  | +0.0629±0.1779 | +0.0205±0.1358 |  |
| Exposure×TIME, *γ_1a_* |  | -0.0157±0.0353 | +0.0413±0.0272 |  |
| DS-B |  |  |  |  |
| Exposure, *γ_0a_* |  | -0.2297±0.1626 | +0.1063±0.1242 |  |
| Exposure×TIME, *γ_1a_* |  | +0.0225±0.0376 | +0.0104±0.0290 |  |
| CDT |  |  |  |  |
| Exposure, *γ_0a_* |  | +0.1308±0.1102 | +0.0595±0.0845 |  |
| Exposure×TIME, *γ_1a_* |  | -0.0409±0.0286 | -0.0308±0.0225 |  |
| TRAILS A |  |  |  |  |
| Exposure, *γ_0a_* |  | +0.6083±4.1689 | -0.7294±3.1572 |  |
| Exposure×TIME, *γ_1a_* |  | +0.7067±1.1793 | -0.3694±0.9284 |  |
| TRAILS B |  |  |  |  |
| Exposure, *γ_0a_* |  | +7.3265±14.2502 | -0.0776±10.803 |  |
| Exposure×TIME, *γ_1a_* |  | -2.6859±3.6267 | +0.7162±2.8222 |  |
| **African American men** |  | **(N=411, k=1.7)** | **(N=411, k=1.7)** |  |
| *Outcome=Cognitive performance test score* |  |  |  |  |
| MMSE |  |  |  |  |
| Exposure, *γ_0a_* |  | +1.1443±1.4227 | -0.9631±1.0792 |  |
| Exposure×TIME, *γ_1a_* |  | -0.8339±0.3925* | +0.2758±0.2964 |  |
| CVLT-List A |  |  |  |  |
| Exposure, *γ_0a_* |  | +0.1645±0.6484 | -0.3087±0.4955 |  |
| Exposure×TIME, *γ_1a_* |  | +0.0617±0.1761 | -0.0172±0.1349 |  |
| CVLT-DFR |  |  |  |  |
| Exposure, *γ_0a_* |  | -0.1842±0.3082 | -0.1301±0.2378 |  |
| Exposure×TIME, *γ_1a_* |  | +0.0871±0.0828 | +0.0685±0.0638 |  |
| BVRT |  |  |  |  |
| Exposure, *γ_0a_* |  | -0.3697±0.5221 | +0.0296±0.3916 |  |
| Exposure×TIME, *γ_1a_* |  | -0.0995±0.1279 | +0.0068±0.0968 |  |
| BTA |  |  |  |  |
| Exposure, *γ_0a_* |  | +0.0872±0.2292 | -0.0367±0.1746 |  |
| Exposure×TIME, *γ_1a_* |  | +0.0566±0.0597 | -0.0003±0.0460** |  |
| AF |  |  |  |  |
| Exposure, *γ_0a_* |  | -0.2533±0.5559 | +0.2310±0.4193 |  |
| Exposure×TIME, *γ_1a_* |  | -0.1464±0.1126 | +0.0203±0.0854 |  |
| DS-F |  |  |  |  |
| Exposure, *γ_0a_* |  | -0.0495±0.2210 | +0.1815±0.1660 |  |
| Exposure×TIME, *γ_1a_* |  | +0.0389±0.0461 | +0.0423±0.0352 |  |
| DS-B |  |  |  |  |
| Exposure, *γ_0a_* |  | +0.2692±0.2017 | -0.0043±0.1530 |  |
| Exposure×TIME, *γ_1a_* |  | -0.0625±0.0468 | -0.0374±0.0358 |  |
| CDT |  |  |  |  |
| Exposure, *γ_0a_* |  | -0.0532±0.1294 | -0.1824±0.0983 |  |
| Exposure×TIME, *γ_1a_* |  | +0.0624±0.0347 | +0.0554±0.0261* |  |
| TRAILS A |  |  |  |  |
| Exposure, *γ_0a_* |  | -4.6105±4.9577 | -0.2365±3.7343 |  |
| Exposure×TIME, *γ_1a_* |  | +1.9057±2.0614 | +0.577±1.5350 |  |
| TRAILS B |  |  |  |  |
| Exposure, *γ_0a_* |  | -12.588±16.9217 | -16.3268±12.6804 |  |
| Exposure×TIME, *γ_1a_* |  | +2.1377±3.7492 | +5.0116±2.7670 |  |
|  |  |  |  |  |

Abbreviations*:* AF=Animal Fluency; *APOE*=Apolipoprotein E genotype; BMI=Body Mass Index; BTA=Brief Test of Attention; BVRT=Benton Visual Retention Test; CDT=Clock Drawing Test; CES-D=Center for Epidemiologic Studies-Depression; CVLT-DFR=California Verbal Learning Test-Delayed Free Recall; CVLT-List A=California Verbal Learning Test-List A; DS-B=Digits Span-Backward; DS-F=Digits Span-Forward; HANDLS = Healthy Aging in Neighborhood of Diversity across the Lifespan; HEI-2010=Healthy Eating Index, 2010 version; HS = High school; MMSE=Mini-Mental State Examination; SD=Standard Deviation; TRAILS A=Trailmaking Test, Part A; TRAILS B=Trailmaking Test, part B; WRAT-3 = Wide Range Achievement Test, 3rd revision; X = mean.

^a^ Model 2 included each of *APOE2* or *APOE4* allelic dosages, separately as the main predictor for v1 cognitive performance and cognitive change over time (11 test scores), using a series of mixed-effects linear regression models, stratifying by sex and race,. These models adjusted for age, sex, race, poverty status, and the inverse mills ratio, and additionally for educational attainment, the WRAT-3 score, current drug use, current tobacco use, body mass index, self-rated health, co-morbidity index, HEI-2010, total energy intake, and the CES-D total score.

**p* < 0.05** *p* < 0.01; *** *p* < 0.001, test for null hypothesis of γ=0. Bolded values passed correction for multiple testing.
