# Supplementary figures and images for: Race, APOE genotypes, and cognitive decline among middle-aged urban adults
Source: Alzheimers Res Ther. 2021 Jun 30;13:120. doi: 10.1186/s13195-021-00855-y (PMC8247163; doi:10.1186/s13195-021-00855-y)

## Slide 1
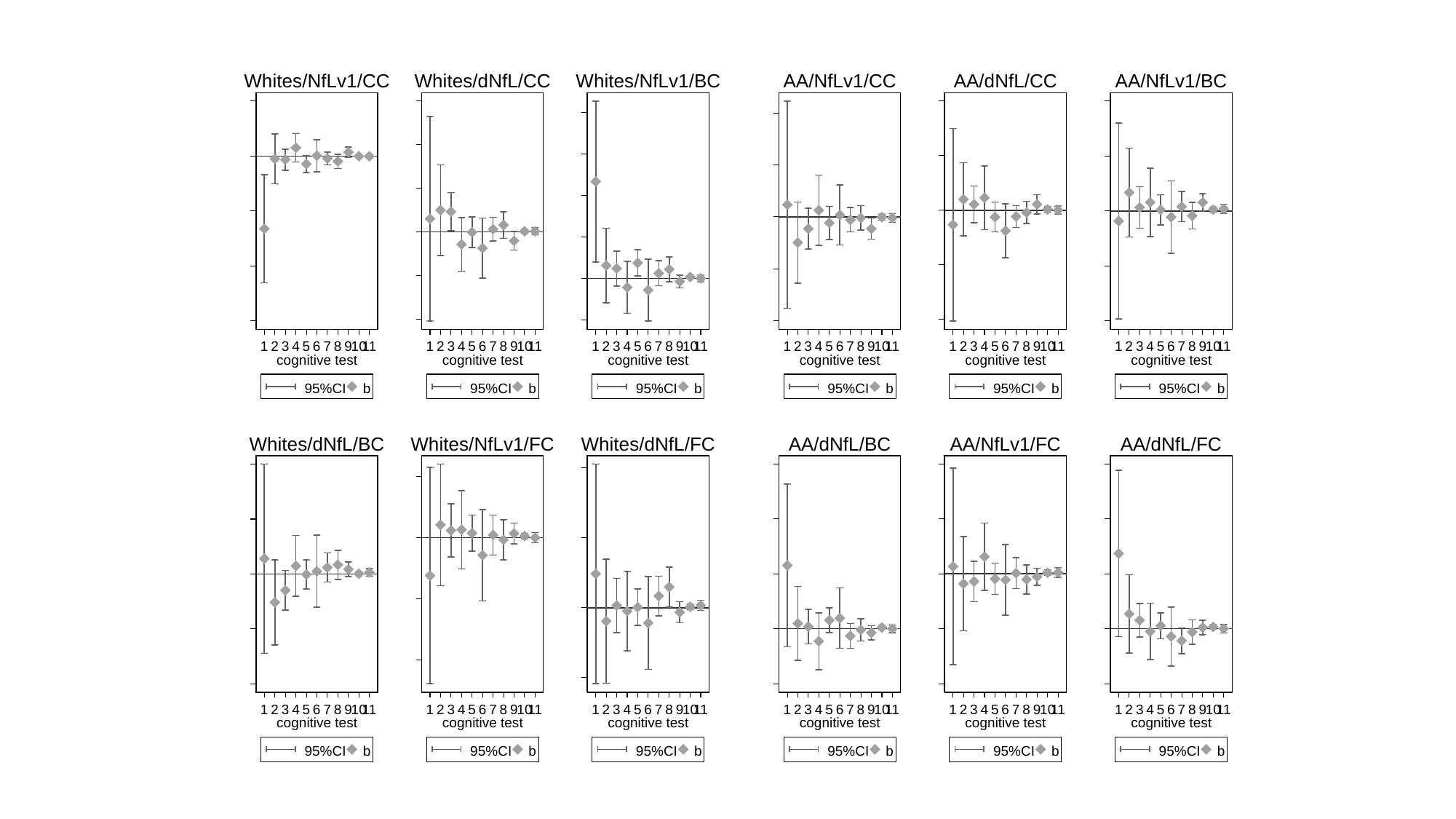

Supplement: Supplementary file 2 — Additional file 2: Figure S1. Standard deviations (SD) of cognitive performance test scores over period of follow-up for each of 11 cognitive tests, in eligible sample (N = 1,770, k = 1.7 observations/participant), HANDLS 2004-2013a. Abbreviations: AF = Animal Fluency; BTA = Brief Test of Attention; BVRT = Benton Visual Retention Test; CDT = Clock Drawing Test; CVLT-DFR = California Verbal Learning Test-Delayed Free Recall; CVLT-List A = California Verbal Learning Test-List A; DS-B=Digits Span-Backward; DS-F=Digits Span-Forward; HANDLS = Healthy Aging in Neighborhood of Diversity across the Lifespan; MMSE = Mini-Mental State Examination; SD=Standard Deviation; TRAILS A = Trailmaking Test, Part A; TRAILS B = Trailmaking Test, part B. a SD computed for each of 11 cognitive test performances over v1 and/or v2 for eligible sample (N = 1,770, k = 1.7 observations/participant). The SD is used to contextualize the effects obtained in mixed-effects linear regression models as a proportion of the SD. [file 13195_2021_855_MOESM2_ESM.pptx]
